# Correction: Silva Julian et al. Severe COVID-19 Outcomes in Five Latin American Countries in the Postvaccination Era. *Viruses* 2024, *16,* 1025

**DOI:** 10.3390/v16101590

**Published:** 2024-10-10

**Authors:** Guilherme Silva Julian, Júlia Spinardi, Melissa Diaz-Puentes, Diana Buitrago, Ida Caterina García, Moe H. Kyaw

**Affiliations:** 1 Evidence Generation Medical Affairs, Pfizer Inc., São Paulo 04717-904, Brazil; 2 Vaccines Medical Affairs, Pfizer Inc., São Paulo 04717-904, Brazil; julia.spinardi@pfizer.com; 3 Faculdade de Ciências Médicas da Santa Casa de São Paulo, São Paulo 01224-001, Brazil; 4 Real World Insights (RWI), IQVIA, Bogotá 110110, Colombia; dianacamila.buitrago@iqvia.com; 5 Real World Insights (RWI), IQVIA, Ciudad de México C.P. 03810, Mexico; idacaterina.garcia@iqvia.com; 6 Vaccines Clinical Epidemiologist Emerging Markets, Pfizer Inc., Collegeville, PA 19426-3982, USA; moe.kyaw@pfizer.com

## Text Correction

In the original publication [1], we identified an error in the reported number of hospitalized cases in Brazil. The error occurred due to the incorrect application of a filter to the dataset, which inadvertently included all cases of severe acute respiratory infection (SARI) rather than exclusively those with laboratory-confirmed COVID-19. Consequently, we reported 18,395,237 hospitalized cases in Brazil in our publication, whereas the correct number, which includes only confirmed COVID-19 cases, is 17,751,803. A correction has been made to the following sections: the Abstract; Section 3. Results; 3.1.1. Hospitalizations; 3.1.4. Deaths; 3.2. Vaccination; and 3.3. Incidence Rates by Variant.

Corrected Abstract:

We conducted a multicountry retrospective study using data from COVID-19 national surveillance databases to analyze clinical profiles, hospitalization rates, intensive care unit (ICU) admissions, utilization of ventilatory support, and mortality rates in five Latin American countries in the context of COVID-19 vaccination implementation. We analyzed the sociodemographic characteristics, comorbidities, clinical outcomes, and vaccination status of laboratory-confirmed COVID-19 cases from January 2021 to December 2022. We calculated the yearly and quarterly hospitalization rates per 1000 confirmed COVID-19 cases and ICU admissions, use of mechanical ventilators, and mortality rates per 1000 hospitalized cases, with their corresponding 95% confidence interval (CI) of 38,209,397 confirmed COVID-19 cases. Rates of hospitalization, ICU admission, ventilatory support, and death were higher among males than among females (30.6 vs. 25, 275.9 vs. 218.8, 156.4 vs. 118.6, and 388.4 vs. 363.1 per 1000, respectively); higher in 2021 than in 2022 (51.6 vs. 20.2, 471.4 vs. 75.5, 230.1 vs. 46.7, and 307.9 vs. 230.3 per 1000, respectively); and higher in the >50 age group (range: 4.3–16.3, 35.5–149.5, 20.1–83.2, and 315–462.9, per 1000) than the <50 age group (range: 0.8–5.7, 3.0–49.3, 2.1–39.3, and 7.8–217.7 per 1000). Hypertension and diabetes mellitus were the most common comorbidities in Mexico and Colombia. Prevention and treatment strategies for these case profiles could bring benefits from a public health perspective.

Corrected paragraphs in Section 3. Results:

A total of 46,176,672 COVID-19-confirmed cases were retrieved across countries’ surveillance databases. After excluding cases with duplicate or missing information (Supplementary Figure S2), our study included 38,209,397 confirmed COVID-19 cases, of which 45.7% were from Brazil, 14.2% were from Mexico, 12.2% were from Colombia, 18.7% were from Argentina, and 8.5% were from Chile, in the period 2021–2022. 

In the overall population, the proportion of female COVID-19 cases (55.1%) exceeds that of males, and most cases were between 18 and 49 years old (62.3%) (Table 1). In Brazil, 37.4% of all confirmed cases were reported among vaccinated individuals, whereas in Colombia, the corresponding percentage was higher, at 74.4%. In the aggregate data from all five countries, 2,122,629 cases required hospitalization (5.6%), and 769,941 died (2.3%) between 2021 and 2022. The percentage of cases who needed ventilatory support was 2.9% (all countries with available data), and 1.3% of the cases needed ICU admission (all countries with available data). 

The age and sex distributions in each country were similar to the overall study population. Fatal outcomes occurred in an average of 2.3% of the confirmed cases in all countries, ranging from 0.2% in Chile to 2.9% in Mexico. The percentage of cases that needed hospitalization ranged from 1.6% in Chile to 7.4% in Brazil. The proportion of cases who needed ventilatory support and ICU admission was also higher in Brazil (5.3% and 2.5%, respectively) than in other countries (below 1%).

Corrected paragraph in Section 3.1.1. Hospitalizations:

Incidence rates were greater for male COVID-19 cases (30.6 per 1000 confirmed cases) than for female cases (25.0 per 1000 confirmed cases), and this trend was observed across all countries for the entire study period (Table 2). The age group with the greatest hospitalization rates was the 50–64 age group in all countries (21.4 for Brazil, 19.5 for Mexico, 11.3 for Colombia, and 4.7 for Argentina), except for Chile, where higher rates were observed in cases who were in the 71–80 age group (3.2 per 1000 confirmed cases). Hospitalization rates decreased from 51.6 in 2021 to 20.2 in 2022 overall, and in all countries individually except for Mexico (Table 3 and Supplementary Figure S8). In Mexico and Colombia, the prevailing comorbidities among hospitalized cases consistently included hypertension (35.3% and 32.3%, respectively) and diabetes mellitus (DM) (29.6% and 13.9%, respectively). Decompensated chronic respiratory diseases (35.5%), carriers of chromosomal diseases or immunologically fragile states (35.4%), and hematologic disease (35.1%) were the most reported comorbidities in Brazil (Supplementary Table S3).

Corrected paragraph in Section 3.1.4. Deaths:

Mortality rates were higher among males than among females (388.4 vs. 363.1 per 1000 hospitalized cases) and increased with age in all countries (Table 4). Overall, mortality decreased over time in all countries except Mexico, where rates remained constant throughout the whole study period (Supplementary Figure S9).

Corrected paragraph in Section 3.2. Vaccination:

Across all age groups, the proportion of unvaccinated individuals was highest among deceased cases, followed by hospitalized and non-hospitalized cases (Figure 1). In Brazil, the proportion of unvaccinated cases was 70.5% among those requiring hospitalization (Supplementary Table S3), 71.3% among cases needing ICU admission (Supplementary Table S5), 70.4% among cases requiring ventilatory support (Supplementary Table S4), and 71.1% among cases with fatal outcomes (Supplementary Table S6). In Colombia, 42.6% of cases requiring hospital admission and 68.9% of cases with a fatal outcome were unvaccinated (Supplementary Tables S3 and S6).

Corrected paragraph in Section 3.3. Incidence Rates by Variant:

In 2021, the 20J Gamma V3 variant predominated in Brazil (in Q1, Q2, and Q3), Mexico (in Q3 and Q4), and Argentina (in Q2 and Q3), coinciding with elevated rates of hospitalization (23.0, 15.7, and 9.0 per 1000 confirmed cases, respectively), ventilatory support (232.5, 19.8, and 79.9 per 1000 confirmed cases, respectively), and ICU admission (106.7 and 13.1 per 1000 confirmed cases, respectively) (Tables 3 and 4 and Supplementary Figures S8 and S9). The same year, the 21H Mu variant was predominant in Colombia from Q1 to Q3, correlating with the highest hospitalization rate (17.9 per 1000 confirmed cases) in Q2.

In 2022, Omicron was present in all countries. The 22B Omicron variant was the most frequent in Brazil (in Q3 and Q4) and Mexico (in Q3 and Q4), coinciding with the lowest reported rates of hospitalization (1.8 and 2.9 per 1000 confirmed cases, respectively), ventilatory support (13.7 and 1.9 per 1000 confirmed cases, respectively), and ICU admission (7.3 and 3.2 per 1000 confirmed cases, respectively) in Q4. Colombia’s most frequent variant was 22A Omicron in Q3 and Q4, coinciding with the lowest hospitalization rate (0.6 per 1000 confirmed cases) in Q4. In Argentina, multiple Omicron sublineages were present throughout the year (21K, 21L, 22B, and 22E), with the lowest rates of hospitalization (0.2 per 1000 confirmed cases), ventilatory support (0.6 per 1000 confirmed cases), and ICU admission (1.6 per 1000 confirmed cases) observed in Q2 (Tables 2 and 3, and Supplementary Table S2).

## Error in Tables and Figures

Tables 1–4 and Figure 1 have also been updated for the same reason. The corrected Table 1, Table 2, Table 3 and Table 4 and Figure 1 appear below. 

**Table 1:** Characteristics of the study population (confirmed COVID-19 cases per country).

	Brazil	Mexico	Colombia	Argentina	Chile	Total
	(*n* = 17,751,803)	(*n* = 5,427,997)	(*n* = 4,644,148)	(*n* = 7,131,436)	(*n* = 3,254,013)	(*n* = 38,209,397)
Percent of participants	45.7%	14.2%	12.2%	18.7%	8.5%	100.0%
Confirmed COVID-19 rate per 1000 individuals	82.6	28.2	156.1	154.2	-	-
Surveillance period	January 2021–December 2022	January 2021–December 2022	January 2021–December 2022	January 2021– June 2022	October 2021–December 2022	-
Sex, *n* (%)						
Female	9,834,763 (55.4)	2,953,304 (54.4)	2,440,147 (52.5)	3,721,818 (52.2)	2,119,577 (54.2)	2,1069,609 (55.1)
Male	7,916,338 (44.6)	2,474,693 (45.6)	2,195,856 (47.3)	3,386,340 (47.5)	1,788,553 (45.8)	17,761,780 (46.5)
Missing	702 (0.0)	0 (0)	0 (0)	23,278 (0.3)	0 (0)	23,980 (0.1)
Age group, *n* (%)						
0–4 years	339,244 (1.9)	60,385 (1.1)	95,500 (2.1)	51,908 (0.7)	-	547,037 (1.6) a
5–17 years	1,213,990 (6.8)	332,247 (6.1)	334,631 (7.2)	435,339 (6.1)	-	2,316,207 (6.6) a
18–29 years	3,459,429 (19.5)	1,345,869 (24.8)	1,044,569 (22.5)	1,747,598 (24.5)	-	7,597,465 (21.7) a
30–39 years	3,635,229 (20.5)	1,269,871 (23.4)	1,008,790 (21.7)	1,654,993 (23.2)	-	7,568,883 (21.7) a
40–49 years	3,419,927 (19.3)	1,047,737 (19.3)	789,303 (17)	1,366,862 (19.2)	-	6,623,829 (18.9) a
50–64 years	1,140,200 (6.4)	956,156 (17.6)	872,746 (18.8)	1,208,934 (17)	-	4,178,036 (12) a
65–74 years	239,098 (1.3)	257,761 (4.7)	291,481 (6.3)	409,769 (5.7)	-	1,198,109 (3.4) a
75–84 years	173,128 (1.0)	117,057 (2.2)	145,466 (3.1)	184,836 (2.6)	-	620,487 (1.8) a
85+ years	99,387 (0.6)	40,912 (0.8)	61,605 (1.3)	69,845 (1)	-	271,749 (0.8) a
Missing	4,032,171 (22.7)	2 (0)	57 (0)	1352 (0)	-	4,033,582 (11.5) a
3–5 years	-	-	-	-	56,541 (1.4)	56,541 (1.4) b
6–11 years	-	-	-	-	174,800 (4.5)	174,800 (4.5) b
12–20 years	-	-	-	-	346,671 (8.9)	346,671 (8.9) b
21–30 years	-	-	-	-	617,741 (15.8)	617,741 (15.8) b
31–40 years	-	-	-	-	652,629 (16.7)	652,629 (16.7) b
41–50 years	-	-	-	-	495,282 (12.7)	495,282 (12.7) b
51–60 years	-	-	-	-	416,448 (10.7)	416,448 (10.7) b
61–70 years	-	-	-	-	278,912 (7.1)	278,912 (7.1) b
71–80 years	-	-	-	-	142,332 (3.6)	142,332 (3.6) b
80+ years	-	-	-	-	72,657 (1.9)	72,657 (1.9) b
Missing	-	-	-	-	654,117 (16.7)	654,117 (16.7) b
Patients who needed hospitalization, *N* (%)	1,307,618 (7.4)	375,146 (6.9)	195,321 (4.2)	128,373 (1.8)	116,171 (1.6)	2,122,629 (5.6)
Hospitalization rate per 1000 Confirmed COVID-19 cases	73.7	69.1	42.1	18.0	35.7	-
Patients who needed ventilatory support, *N* (%)	938,850 (5.3)	33,612 (0.6)	1515 (0)	18,728 (0.3)	-	992,705 (2.9) a
Ventilatory support rate per 1000 hospitalized COVID-19 cases	718.0	89.6	7.8	145.9	-	-
Patients who needed ICU admission, *N* (%)	443,359 (2.5)	25,572 (0.5)	-	31,236 (0.4)	7868 (0.1)	508,035 (1.3) c
ICU admission rate per 1000 hospitalized COVID-19 cases	339.1	68.2	-	243.3	67.7	-
Patients who had a fatal outcome, N (%)	424,606 (2.4)	158,298 (2.9)	94,354 (2)	79,615 (1.1)	13,068 (0.2)	769,941 (2.3)
Mortality rate of COVID-19 patients per 1000 hospitalized cases	324.7	422.0	483.1	620.2	112.5	-

a, Percentages in the total number of cases excluding Chile; b, percentages in the total number of cases in Chile; c, percentages in the total number of cases excluding Colombia.

**Table 2:** Hospitalization rates per 1000 confirmed COVID-19 cases.

	Brazil		Mexico		Colombia		Argentina		Chile		Total	
	(*n* = 1,307,618)		(*n* = 375,146)		(*n* = 195,321)		(*n* = 128,373)		(*n* = 116,171)		(*n* = 2,122,629)	
	Rate	95% CI	Rate	95% CI	Rate	95% CI	Rate	95% CI	Rate	95% CI	Rate	95% CI
Percent of participants	61.1%		17.7%		9.2%		6.0%		5.5%		100.0%	
Surveillance period	January 2021–December 2022		January 2021–December 2022		January 2021–December 2022		January 2021–June 2022		October 2021–December 2022			
Sex												
Female	33.2	33.1–33.3	30.8	30.7–30.9	18	17.9–18.1	7.9	7.8–8	17.5	17.4–17.6	25	24.9–25.1
Male	40.5	40.4–40.5	38.4	38.2–38.6	24	23.9–24.1	9.9	9.8–10	18.2	18.1–18.3	30.6	30.5–30.7
Age group												
0–4 years	1.2	1.2–1.3	1.4	1.4–1.4	1.8	1.8–1.8	0.3	0.3–0.3	-	-	1.1	1.1–1.1
5–17 years	0.7	0.7–0.7	1.5	1.5–1.5	0.7	0.7–0.7	0.4	0.4–0.4	-	-	0.8	0.7–0.8
18–29 years	3.1	3.1–3.2	4.4	4.3–4.5	1.9	1.9–1.9	0.8	0.8–0.8	-	-	2.7	2.7–2.7
30–39 years	7.8	7.7–7.8	6.7	6.6–6.8	3.1	3–3.2	1.3	1.3–1.3	-	-	5.7	5.7–5.7
40–49 years	11.6	11.6–11.7	9.4	9.3–9.5	4.7	4.6–4.8	2.2	2.2–2.2	-	-	8.4	8.4–8.4
50–64 years	21.4	21.3–21.4	19.5	19.4–19.6	11.3	11.2–11.4	4.7	4.6–4.8	-	-	16.3	16.3–16.4
65–74 years	13	12.9–13.0	13.1	13–13.2	8.1	8–8.2	3.5	3.5–3.5	-	-	10.4	10.4–10.4
75–84 years	9.4	9.4–9.5	9.1	9–9.2	6.7	6.6–6.8	2.9	2.9–2.9	-	-	7.7	7.7–7.7
85+ years	5.4	5.4–5.4	4	3.9–4.1	3.8	3.7–3.9	1.9	1.9–1.9	-	-	4.3	4.2–4.3
3–5 years	-	-	-	-	-	-	-	-	0.1	0.1–0.1	0.1	0.1–0.1
6–11 years	-	-	-	-	-	-	-	-	0.2	0.2–0.2	0.2	0.2–0.2
12–20 years	-	-	-	-	-	-	-	-	0.5	0.5–0.5	0.5	0.5–0.5
21–30 years	-	-	-	-	-	-	-	-	1.2	1.2–1.2	1.2	1.2–1.2
31–40 years	-	-	-	-	-	-	-	-	1.4	1.4–1.4	1.4	1.4–1.4
41–50 years	-	-	-	-	-	-	-	-	1.3	1.3–1.3	1.3	1.3–1.3
51–60 years	-	-	-	-	-	-	-	-	2.0	2–2	2.0	2–2
61–70 years	-	-	-	-	-	-	-	-	2.8	2.7–2.9	2.8	2.7–2.9
71–80 years	-	-	-	-	-	-	-	-	3.2	3.1–3.3	3.2	3.1–3.3
80+ years	-	-	-	-	-	-	-	-	3.1	3–3.2	3.1	3–3.2
Period												
2021Q1	23	23.0–23.1	5.5	5.4–5.6	9.5	9.4–9.6	3.0	3–3	-	-	14.4	14.4–14.4
2021Q2	20.8	20.7–20.8	1.8	1.8–1.8	17.9	17.8–18	9.0	8.9–9.1	-	-	15.1	15.1–15.1
2021Q3	10.3	10.3–10.4	15.7	15.6–15.8	5.2	5.1–5.3	2.7	2.7–2.7	-	-	8.9	8.9–8.9
2021Q4	7.9	7.9–8.0	9.5	9.4–9.6	2.0	2–2	0.5	0.5–0.5	-	-	5.9	5.9–5.9
2022Q1	5.1	5.1–5.2	16.6	16.5–16.7	5.0	4.9–5.1	2.6	2.6–2.6	-	-	6.4	6.4–6.4
2022Q2	2.6	2.6–2.7	6.4	6.3–6.5	0.8	0.8–0.8	0.2	0.2–0.2	-	-	2.5	2.5–2.5
2022Q3	1.8	1.7–1.8	10.0	9.9–10.1	1.2	1.2–1.2	-	-	-	-	2.6	2.6–2.6
2022Q4	2.1	2.1–2.1	2.9	2.9–2.9	0.6	0.6–0.6	-	-	-	-	1.6	1.6–1.6
Year												
2021	62.1	62.0–62.2	32.5	32.3–32.7	34.6	34.4–34.8	15.1	15–15.2	14.8	14.7–14.9	51.6	51.5–51.7
2022	11.6	11.5–11.6	35.8	35.6–36	7.5	7.4–7.6	2.8	2.8–2.8	20.9	20.7–21.1	20.2	20.2–20.2

Rates among the total number of confirmed COVID-19 cases in countries with available information.

**Table 3:** Ventilatory support and ICU admission rates per 1000 hospitalized COVID-19 cases.

	Brazil		Mexico		Colombia		Argentina		Chile		Total	
	Rate	95% CI	Rate	95% CI	Rate	95% CI	Rate	95% CI	Rate	95% CI	Rate	95% CI
Ventilatory Support	(*n* = 938,850)		(*n* = 33,612)		(*n* = 1515)		(*n* = 18,728)		-		(*n* = 992,705)	
Percent of participants	94.6%		3.4%		0.2%		1.9%		0.00%		100.00%	
Surveillance period	1/2021–12/2022		1/2021–12/2022		1/2021–12/2022		1/2021–6/2022		-		-	
Sex												
Female	319.8	319.0–320.6	34.9	34.3–35.5	3.3	3–3.6	54.5	53.2–55.8	-	-	218.8 a	218.2–219.4
Male	398.1	397.3–399.0	54.7	54–55.4	4.5	4.2–4.8	90.7	89.1–92.3	-	-	275.9 a	275.2–276.6
Age group												
0–4 years	7.8	7.7–8.0	1.1	1–1.2	0.1	0.1–0.1	0.6	0.5–0.7	-	-	5.4 a	5.3–5.5
5–17 years	4.3	4.2–4.4	0.9	0.8–1	0	0–0	0.9	0.7–1.1	-	-	3 a	2.9–3.1
18–29 years	26	25.7–26.3	3.2	3–3.4	0.2	0.1–0.3	3.2	2.9–3.5	-	-	17.8 a	17.6–18
30–39 years	72.7	72.3–73.2	7.1	6.8–7.4	0.5	0.4–0.6	8	7.5–8.5	-	-	49.3 a	49–49.6
40–49 years	113.8	113.3–114.3	12.7	12.3–13.1	1.2	1–1.4	18.3	17.6–19	-	-	77.8 a	77.4–78.2
50–64 years	215.6	214.9–216.3	29.2	28.7–29.7	2.8	2.6–3	50.7	49.5–51.9	-	-	149.5 a	149–150
65–74 years	131	130.4–131.6	19.7	19.3–20.1	1.7	1.5–1.9	37.3	36.2–38.4	-	-	91.6 a	91.2–92
75–84 years	93.6	93.1–94.1	11.9	11.6–12.2	1.1	1–1.2	21	20.2–21.8	-	-	64.7 a	64.3–65.1
85+ years	52.8	52.5–53.2	3.8	3.6–4	0.2	0.1–0.3	5.8	5.4–6.2	-	-	35.5 a	35.2–35.8
Period												
2021Q1	232.5	231.8–233.3	9.5	9.2–9.8	-	-	23.3	22.5–24.1	-	-	171.5 b	170.9–172.1
2021Q2	211.6	210.9–212.3	2.7	2.5–2.9	-	-	79.9	78.4–81.4	-	-	159 b	158.4–159.6
2021Q3	103.6	103.1–104.1	19.8	19.3–20.3	-	-	23	22.2–23.8	-	-	80.5 b	80.1–80.9
2021Q4	78.9	78.5–79.4	15.3	14.9–15.7	-	-	3.7	3.4–4	-	-	60.4 b	60–60.8
2022Q1	41.7	41.3–42.0	17.3	16.9–17.7	-	-	15.5	14.8–16.2	-	-	34.7 b	34.4–35
2022Q2	20.3	20.0–20.5	8.1	7.8–8.4	-	-	0.6	0.5–0.7	-	-	16.4 b	16.2–16.6
2022Q3	13.7	13.5–13.9	13.5	13.1–13.9	-	-	-		-	-	12.7 b	12.5–12.9
2022Q4	15.7	15.5–16.0	1.9	1.8–2	-	-	-		-	-	11.8 b	11.6–12
Year												
2021	626.6	625.8–627.4	47.3	46.6–48	-	-	129.2	127.2–131.2	-	-	471.4 b	470.4–472.4
2022	91.4	90.9–91.9	40.7	40.1–41.3	-	-	16	15.3–16.7	-	-	75.5 b	75.1–75.9
ICU admission	(*n* = 443,359)		(*n* = 25,572)		-		(*n* = 31,236)		(*n* = 7868)		(*n* = 508,035)	
Percent of participants	87.3%		5.0 %		0.00%		6.1%		1.5%		100.00%	
Surveillance period	1/2021–12/2022		1/2021–12/2022		1/2021–12/2022		1/2021–6/2022		10/2021–12/2022		-	
Sex												
Female	147.7	147.1–148.3	27.5	27–28	-	-	95.1	93.4–96.8	-	-	119.1 a	118.6–119.6
Male	191.3	190.6–192.0	40.7	40.1–41.3	-	-	146.8	144.7–148.9	-	-	157 a	156.4–157.6
Age group												00
0–4 years	4.2	4.1–4.3	1.5	1.4–1.6	-	-	1.5	1.3–1.7	-	-	3.4 a	3.3–3.5
5–17 years	2.4	2.3–2.5	1	0.9–1.1	-	-	2.1	1.8–2.4	-	-	2.1 a	2–2.2
18–29 years	10.9	10.7–11.1	3.4	3.2–3.6	-	-	6.5	6.1–6.9	-	-	9 a	8.9–9.1
30–39 years	29.7	29.4–30.0	6.5	6.2–6.8	-	-	14.4	13.7–15.1	-	-	23.8 a	23.6–24
40–49 years	48.6	48.3–49.0	9.9	9.6–10.2	-	-	29.6	28.7–30.5	-	-	39.3 a	39–39.6
50–64 years	101.2	100.7–101.7	20.5	20–21	-	-	77.6	76.1–79.1	-	-	82.8 a	82.4–83.2
65–74 years	68.5	68.0–68.9	13	12.6–13.4	-	-	58.9	57.6–60.2	-	-	56.3 a	56–56.6
75–84 years	48.4	48.1–48.8	8.8	8.5–9.1	-	-	37.8	36.7–38.9	-	-	39.5 a	39.2–39.8
85+ years	25	24.7–25.2	3.7	3.5–3.9	-	-	15	14.2–15.6	-	-	19.9 a	19.7–20.1
3–5 years	-	-	-	-	-	-	-	-	0.4	0.3–0.5	0.4 b	0.3–0.5
6–11 years	-	-	-	-	-	-	-	-	0.5	0.4–0.6	0.5 b	0.4–0.6
12–20 years	-	-	-	-	-	-	-	-	1	0.8–1.2	1 b	0.8–1.2
21–30 years	-	-	-	-	-	-	-	-	2.4	2.1–2.7	2.4 b	2.1–2.7
31–40 years	-	-	-	-	-	-	-	-	4.7	4.3–5.1	4.7 b	4.3–5.1
41–50 years	-	-	-	-	-	-	-	-	6.5	6.0–7.0	6.5 b	6.0–7.0
51–60 years	-	-	-	-	-	-	-	-	12.1	11.5–12.7	12.1 b	11.5–12.7
61–70 years	-	-	-	-	-	-	-	-	17.7	16.9–18.5	17.7 b	16.9–18.5
71–80 years	-	-	-	-	-	-	-	-	15.8	15.1–16.5	15.8 b	15.1–16.5
80+ years	-	-	-	-	-	-	-	-	6.6	6.1–7.1	6.6 b	6.1–7.1
Period												
2021Q1	106.7	106.2–107.3	4.5	4.3–4.7	-	-	41.4	40.3–42.5	-	-	80.9 a	80.5–81.3
2021Q2	94.2	93.7–94.7	2	1.9–2.1	-	-	123.6	121.7–125.5	-	-	77.1 a	76.7–77.5
2021Q3	50.6	50.2–50.9	13.1	12.7–13.5	-	-	39.2	38.1–40.3	-	-	42 a	41.7–42.3
2021Q4	38.1	37.8–38.5	6.9	6.6–7.2	-	-	7.1	6.6–7.6	-	-	29.5 a	29.2–29.8
2022Q1	22.5	22.3–22.8	15	14.6–15.4	-	-	30.4	29.4–31.4	-	-	21.5 a	21.3–21.7
2022Q2	10.9	10.8–11.1	9.2	8.9–9.5	-	-	1.6	1.4–1.8	-	-	9.9 a	9.8–10
2022Q3	7.3	7.1–7.4	13.2	12.8–13.6	-	-	-	-	-	-	8 a	7.9–8.1
2022Q4	8.7	8.5–8.9	3.2	3–3.4	-	-	-	-	-	-	6.9 a	6.8–7
Year												
2021	289.6	288.8–290.4	26.4	25.9–26.9	-	-	210	207.5–212.5	-	-	229.4 a	228.7–230.1
2022	49.5	49.1–49.8	40.7	40.1–41.3	-	-	31.9	30.9–32.9	-	-	46.4 a	46.1–46.7

a, Rates among the total number of cases excluding Chile and Colombia; b, rates among the total number of cases in Chile.

**Table 4:** Mortality rates of COVID-19 cases per 1000 hospitalized cases.

	Brazil		Mexico d		Colombia		Argentina		Chile b		Total	
	(*n* = 424,606)		(*n* = 158,298)		(*n* = 94,354)		(*n* = 79,615)		(*n* = 13,068)		(*n* = 769,941)	
	Rate	95% CI	Rate	95% CI	Rate	95% CI	Rate	95% CI	Rate	95% CI	Rate	95% CI
Percent of participants	55.1%		20.6%		12.3%		10.3%		1.7%		100.0%	
Surveillance period	1/2021–12/2022		1/2021–12/2022		1/2021–12/2022		1/2021–6/2022		10/2021–12/2022			
Sex												
Female	322.6	321.4–323.8	382.9	380–385.9	450.4	445.8–454.9	597.2	590.8–603.6	-	-	363.1 a	361.9–364.3
Male	326.4	325.3–327.5	453.3	450.4–456.1	508.4	504.2–512.6	637.4	631.5–643.2	-	-	388.4 a	387.2–389.6
Age group												
0–4 years	45.8	43.1–48.6	54.9	49.6–60.1	14.7	12.1–17.3	43.3	33.8–52.8	-	-	7.8 a	7.4–8.2
5–17 years	70.8	66.3–75.4	55.8	50.6–61	39.2	32.5–45.8	55.0	46.2–63.8	-	-	20.6 a	19.6–21.6
18–29 years	119.4	116.7–122.1	129.7	125.1–134.3	136.8	129.1–144.5	117.6	108.7–126.5	-	-	95.6 a	93.9–97.3
30–39 years	153.2	151.3–155.1	230.3	225.4–235.3	236.8	228.9–244.7	206.9	197.8–216.1	-	-	152.5 a	150.9–154.1
40–49 years	207.3	205.5–209.0	333.5	328.5–338.5	347.9	340–355.7	336.4	327.3–345.5	-	-	217.7 a	216.1–219.3
50–64 years	310.1	308.6–311.5	452.4	448.4–456.5	464.7	458.9–470.5	539.2	531.3–547	-	-	315 a	313.6–316.4
65–74 years	434.3	432.3–436.3	550.1	544.7–555.6	592.7	584.9–600.5	794.3	783.3–805.3	-	-	406.8 a	404.9–408.7
75–84 years	490.9	488.5–493.3	583.2	576.5–590	672.5	663.3–681.6	922.5	909.5–935.6	-	-	434.9 a	432.7–437.1
85+ years	545.3	542.1–548.4	596.7	586.4–606.9	811.3	798.1–824.5	1063.3	1045.7–1080.9	-	-	462.9 a	4599–4659
3–5 years	-	-	-	-	-	-	-	-	12.0	1.5–22.6	12 c	1.5–22.5
6–11 years	-	-	-	-	-	-	-	-	12.9	4–21.9	12.9 c	4–21.8
12–20 years	-	-	-	-	-	-	-	-	17.1	10.9–23.3	17.1 c	10.9–23.3
21–30 years	-	-	-	-	-	-	-	-	19.9	15.5–24.4	19.9 c	15.5–24.3
31–40 years	-	-	-	-	-	-	-	-	35.3	29.8–40.8	35.3 c	29.8–40.8
41–50 years	-	-	-	-	-	-	-	-	89.6	80.4–98.7	89.6 c	80.4–98.8
51–60 years	-	-	-	-	-	-	-	-	149.5	140–159	149.5 c	140–159
61–70 years	-	-	-	-	-	-	-	-	205.7	196.3–215.1	205.7 c	196.3–215.1
71–80 years	-	-	-	-	-	-	-	-	318.8	307.9–329.7	318.8 c	307.9–329.7
80+ years	-	-	-	-	-	-	-	-	624.8	609.4–640.2	624.8 c	609.4–640.2
Period												
2021Q1	360.2	358.7–361.7	32.4	31.9–33	444.0	437.8–450.3	635.2	624.5–646	-	-	193.9 a	193–194.8
2021Q2	305.6	304.1–307.1	8.8	8.5–9.1	568.8	563.7–573.9	661.4	655.1–667.7	-	-	209.8 a	208.9–210.7
2021Q3	316.2	314.0–318.3	29.4	29.1–29.7	498.0	489.1–507	581.1	570.4–591.8	-	-	76.1 a	75.7–76.5
2021Q4	330.7	328.2–333.2	47.1	46.5–47.7	394.2	381.3–407	472.6	449.6–495.6	-	-	100.4 a	99.7–101.1
2022Q1	325.7	322.6–328.7	31.5	31.2–31.8	400.0	391.8–408.2	558.9	548.3–569.6	-	-	63.7 a	63.3–64.1
2022Q2	260.7	256.7–264.7	34.3	33.7–34.8	198.0	183.2–212.8	205.4	180.7–230.1	-	-	50.5 a	49.9–51.1
2022Q3	274.8	269.9–279.8	32.9	32.5–33.3	239.6	226.9–252.4	-	-	-	-	41.6 a	41.2–42
2022Q4	263.7	259.2–268.1	28.4	27.7–29.1	224.5	206.4–242.5	-	-	-	-	53.9 a	53.1–54.7
Year												
2021	330.8	329.9–331.7	434.8	431.7–437.9	514.0	510.5–517.5	635.1	630.3–639.9	-	-	307.9 a	307.1–308.7
2022	292	290.1–294.0	409.0	406.1–411.8	340.3	334.2–346.4	536.3	526.1–546.4	-	-	230.3 a	229.2–231.4

a, Rates among the total number of cases excluding Chile; b, data from week 40 of 2021 to week 51 of 2022; c, rates among the total number of cases in Chile; d, mortality rates among hospitalized COVID-19 cases.

**Figure 1:**
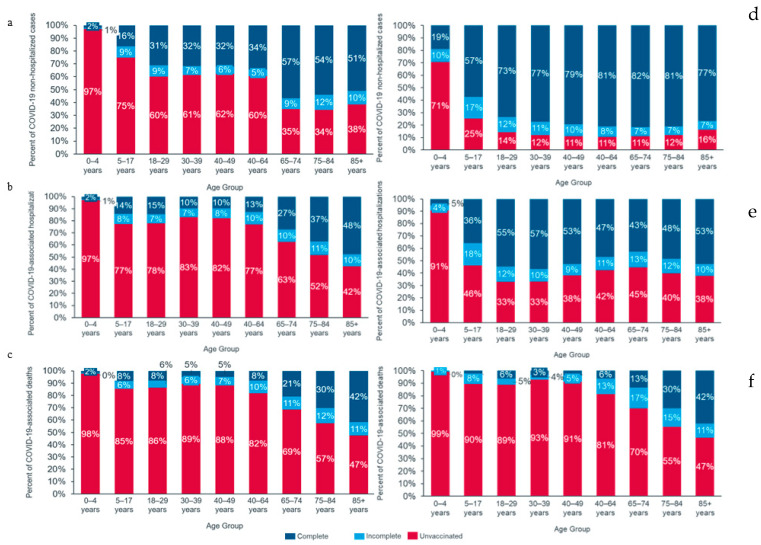
Vaccination status by age group in Brazil and Colombia for non-hospitalized, hospitalized, and deceased COVID-19 patients. Figures (**a**–**c**) correspond to Brazil, and figures (**d**–**f**) correspond to Colombia. Figures (**a**,**d**) correspond to non-hospitalized cases, (**b**,**e**) to COVID-19-associated hospitalizations, and (**c**,**f**) to COVID-19-associated deaths. Bars in red represent the proportion of unvaccinated cases among each age group. Bars in light blue correspond to the proportion of cases with an incomplete COVID-19 primary vaccination schedule and dark blue to the proportion of cases with a complete COVID-19 primary vaccination schedule among each age group. The proportion of unvaccinated cases was the highest among the deceased, followed by hospitalized cases. The proportion of cases with complete vaccination schedules was highest among older age groups for all displayed outcomes.

## Error in Supplementary Materials 

The Supplementary Materials have also been updated for the same reason. The corrected Tables S3–S6, Figures S2–S9 appear below. 

**Table S3:** Characteristics of hospitalized COVID-19 cases per country.

Characteristic	Brazil	Mexico	Colombia	Argentina	Chile	Total
	(*n* = 1,307,618)	(*n* = 375,146)	(*n* = 195,321)	(*n* = 128,373)	(*n* = 116,171)	(*n* = 2,122,629)
**Sex, *n* (%)**						
Female	589,363 (45.1)	166,951 (44.5)	83,383 (42.7)	56,582 (44.1)	57,028 (49.1)	953,307 (44.9)
Male	718,146 (54.9)	208,195 (55.5)	111,687 (57.2)	70,914 (55.2)	59,143 (50.9)	1,168,085 (55)
Missing	109 (0.0)	0 (0)	251 (0.1)	877 (0.7)	0 (0)	1237 (0.1)
**Age groups, *n* (%)**						
0–4 years	22,043 (1.7)	7601 (2.0)	8172 (4.2)	1847 (1.4)	-	39,663 (2) ^b^
5–17 years	12,172 (0.9)	8064 (2.1)	3421 (1.8)	2709 (2.1)	-	26,366 (1.3) ^b^
18–29 years	55,755 (4.3)	23,659 (6.3)	8750 (4.5)	5689 (4.4)	-	93,853 (4.7) ^b^
30–39 years	138,140 (10.6)	36,521 (9.7)	14,413 (7.4)	9535 (7.4)	-	198,609 (9.9) ^b^
40–49 years	205,970 (15.8)	50,898 (13.6)	21,686 (11.1)	15,545 (12.1)	-	294,099 (14.7) ^b^
50–64 years	379,244 (29.0)	105,794 (28.2)	52,613 (26.9)	33,800 (26.3)	-	571,451 (28.5) ^b^
65–74 years	230,153 (17.6)	71,198 (19.0)	37,507 (19.2)	25,098 (19.6)	-	363,956 (18.1) ^b^
75–84 years	167,580 (12.8)	49,594 (13.2)	30,902 (15.8)	20,887 (16.3)	-	268,963 (13.4) ^b^
85+ years	95,981 (7.3)	21,817 (5.8)	17,853 (9.1)	13,197 (10.3)	-	148,848 (7.4) ^b^
Missing	580 (0.0)	0 (0)	4 (0.0)	66 (0.1)	-	650 (0) ^b^
3–5 years	-	-	-	-	415 (0.8) ^c^	415 (0.8) ^d^
6–11 years	-	-	-	-	618 (1.2) ^c^	618 (1.2) ^d^
12–20 years	-	-	-	-	1698 (3.3) ^c^	1698 (3.3) ^d^
21–30 years	-	-	-	-	3914 (7.7) ^c^	3914 (7.7) ^d^
31–40 years	-	-	-	-	4445 (8.7) ^c^	4445 (8.7) ^d^
41–50 years	-	-	-	-	4108 (8.1) ^c^	4108 (8.1) ^d^
51–60 years	-	-	-	-	6394 (12.5) ^c^	6394 (12.5) ^d^
61–70 years	-	-	-	-	8993 (17.6) ^c^	8993 (17.6) ^d^
71–80 years	-	-	-	-	10,313 (20.2) ^c^	10,313 (20.2) ^d^
80+ years	-	-	-	-	10,129 (19.9) ^c^	10,129 (19.9) ^d^
Missing	-	-	-	-	65,144	-
**Period, *n* (%)**						
2021Q1	408,898 (31.3)	29,653 (7.9)	43,982 (22.5)	21,071 (16.4)	-	-
2021Q2	368,765 (28.2)	9874 (2.6)	83,281 (42.6)	64,180 (50.0)	-	-
2021Q3	183,413 (14.0)	85,331 (22.7)	24,070 (12.3)	19,510 (15.20)	-	-
2021Q4	140,706 (10.8)	51,688 (13.8)	9179 (4.7)	3428 (2.70)	-	-
2022Q1	90,900 (7.0)	89,848 (24.0)	23,007 (11.8)	18,889 (14.70)	-	-
2022Q2	46,659 (3.6)	34,745 (9.3)	3485 (1.8)	1295 (1.0)	-	-
2022Q3	31,101 (2.4)	54,065 (14.4)	5671 (2.9)	-	-	-
2022Q4	37,176 (2.8)	15,770 (4.2)	2646 (1.4)	-	-	-
**Year, *n* (%)**						
2021	1,101,782 (84.3)	176,546 (47.1)	160,512 (82.2)	107,413 (83.7)	48,058 (41.4)	1,594,311 (75.1)
2022	205,836 (15.7)	194,428 (51.8)	34,809 (17.8)	20,083 (15.6)	68,113 (58.6)	523,269 (24.7)
Missing	0 (0)	4172 (1.1)	0 (0)	877 (0.7)	0 (0)	5049 (0.2)
**Comorbidities, *n* (%)**						
Chronical cardiac illness	321,316 (24.6)	16,181 (4.3)	-	-	-	-
Hematologic disease	459,122 (35.1)	-	-	-	-	-
Carrier chromosomal disease immunological fragility	463,022 (35.4)	-	-	-	-	-
Hepatic disease	456,462 (34.9)		-	-	-	-
Diabetes	300,120 (23.0)	110,877 (29.6)	27,168 (13.9)	-	-	-
Chronical neurological neuromuscular illness	434,150 (33.2)	-	-	-	-	-
Decompensated chronical respiratory diseases	464,673 (35.5)	-	-	-	-	-
Immunosuppression	443,472 (33.9)	8535 (2.3)	-	-	-	-
Renal disease	436,226 (33.4)	22,630 (6.0)	-	-	-	-
Obesity	383,522 (29.3)	62,268 (16.6)	-	-	-	-
Pregnant	12,775 (2.6)	-	-	-	-	-
Neoplasia/Cancer	7708 (0.6)	-	4900 (2.5)	-	-	-
COPD	-	13,088 (3.5)	-	-	-	-
Asthma	-	6970 (1.9)	-	-	-	-
Hypertension	-	132,337 (35.3)	62,994 (32.3)	-	-	-
Arthritis	-	-	1461 (0.7)	-	-	-
Orphan diseases	-	-	1194 (0.6)	-	-	-
HIV	-	-	739 (0.4)	-	-	-
Smoking	-	24,638 (6.6)	-	-	-	-
Other comorbidities	239,059 (18.3)	17,846 (4.8)	-	-	-	-
**Vaccination status, *n* (%) ^a^**						
Yes	386,288 (29.5)	-	112,147 (57.4)	-	-	498,435 (33.2)
No	921,330 (70.5)	-	83,174 (42.6)	-	-	1,004,504 (66.8)

^a^ ‘Vaccination status = Yes’ when subject has received one or two doses of the initial COVID-19 vaccine (not including booster doses). ^b^ Percentages over the total number of cases excluding Chile. ^c^ Data from week 40 of 2021 to week 51 of 2022. ^d^ Percentages over the total number of cases in Chile.

**Table S4:** Characteristics of COVID-19 cases requiring ventilatory support per country.

Characteristic	Brazil	Mexico	Colombia	Argentina	Chile	Total
	(*n* = 938,850)	(*n* = 33,612)	(*n* = 1515)	(*n* = 18,728)	-	(*n* = 992,705)
**Sex, *n* (%)**						
Female	418,215 (44.5)	13,094 (39.0)	641 (42.3)	6991 (37.3)	-	438,941 (44.2)
Male	520,582 (55.4)	20,518 (61.0)	874 (57.7)	11,642 (62.2)	-	553,616 (55.8)
Missing	53 (0.0)	0 (0)	0 (0)	95 (0.5)	-	148 (0)
**Age groups, *n* (%)**						
0–4 years	10,258 (1.1)	424 (1.3)	17 (1.1)	80 (0.4)	-	10,779 (1.1) ^b^
5–17 years	5666 (0.6)	326 (1.0)	2 (0.1)	117 (0.6)	-	6111 (0.6) ^b^
18–29 years	33,986 (3.6)	1185 (3.5)	37 (2.4)	416 (2.2)	-	35,624 (3.6) ^b^
30–39 years	95,118 (10.1)	2679 (8.0)	101 (6.7)	1021 (5.5)	-	98,919 (10) ^b^
40–49 years	148,808 (15.9)	4766 (14.2)	228 (15.0)	2355 (12.6)	-	156,157 (15.7) ^b^
50–64 years	281,941 (30.0)	10,961 (32.6)	553 (36.5)	6506 (34.7)	-	299,961 (30.2) ^b^
65–74 years	171,281 (18.2)	7384 (22.0)	328 (21.7)	4786 (25.6)	-	183,779 (18.5) ^b^
75–84 years	122,414 (13.0)	4478 (13.3)	208 (13.7)	2700 (14.4)	-	129800 (13.1) ^b^
85+ years	69,086 (7.4)	1409 (4.2)	41 (2.7)	745 (4.0)	-	71,281 (7.2) ^b^
Missing	292 (0.0)	0 (0)	0 (0)	2 (0.0)	-	294 (0) ^b^
**Period, *n* (%)**						
2021Q1	304,068 (32.4)	3560 (10.6)	-	2989 (16.0)	-	310,617 (31.3) ^d^
2021Q2	276,631 (29.5)	1005 (3.0)	-	10,258 (54.8)	-	287,894 (29) ^d^
2021Q3	135,467 (14.4)	7437 (22.1)	-	2950 (15.8)	-	145,854 (14.7) ^d^
2021Q4	103,192 (11.0)	5742 (17.1)	-	473 (2.5)	-	109,407 (11) ^d^
2022Q1	54,470 (5.8)	6475 (19.3)	-	1985 (10.6)	-	62,930 (6.3) ^d^
2022Q2	26,517 (2.8)	3051 (9.1)	-	73 (0.4)	-	29,641 (3) ^d^
2022Q3	17,916 (1.9)	5051 (15.0)	-	-	-	22,967 (2.3) ^d^
2022Q4	20,589 (2.2)	709 (2.1)	-	-	-	21,298 (2.1) ^d^
**Year, *n* (%)**						
2021	819,358 (87.3)	17,744 (52.8)	-	16,580 (88.5)	-	853,682 (86.1)
2022	119,492 (12.7)	15,286 (45.5)	-	2053 (11.0)	-	136,831 (13.8)
Missing	0 (0)	582 (1.7)	-	95 (0.5)	-	9062 (0.9)
**Comorbidities, *n* (%)**						
Chronical cardiac illness	167,217 (27.8)	1679 (5.0)	-	-	-	-
Hematologic disease	250,408 (41.6)	-	-	-	-	-
Carrier chromosomal disease immunological fragility	252,632 (42.0)	-	-	-	-	-
Hepatic disease	249,883 (41.6)		-	-	-	-
Diabetes	163,988 (27.3)	11,134 (33.1)	-	-	-	-
Chronical neurological neuromuscular illness	232,792 (38.7)	-	-	-	-	-
Decompensated chronical respiratory diseases	252,649 (42.0)	-	-	-	-	-
Immunosuppression	241,364 (40.2)	861 (2.6)	-	-	-	-
Renal disease	239,695 (39.9)	1894 (5.6)	-	-	-	-
Obesity	209,745 (34.9)	8194 (24.4)	-	-	-	-
Pregnant	7503 (1.6)	-	-	-	-	-
Neoplasia /Cancer	4012 (0.7)	-	-	-	-	-
COPD	-	1113 (3.3)	-	-	-	-
Asthma	-	655 (1.9)	-	-	-	-
Hypertension	-	13,370 (39.8)	-	-	-	-
Arthritis	-	-	-	-	-	-
Orphan diseases	-	-	-	-	-	-
HIV	-	-	-	-	-	-
Smoking	-	2453 (7.3)	-	-	-	-
Other comorbidities	128,470 (21.4)	1653 (4.9)	-		-	-
**Vaccination status, *n* (%) ^a^**						
Yes	269,084 (28.7) ^c^	-	-	-	-	269,084 (28.7) ^c^
No	669,766 (71.3) ^c^	-	-	-	-	669,766 (71.3) ^c^

^a^ ‘Vaccination status = Yes’ when subject has received one or two doses of the initial COVID-19 vaccine (not including booster doses). ^b^ Percentages over the total number of cases excluding Chile. ^c^ Percentages over the total number of cases in Brazil. ^d^ Percentages over the total number of cases in Brazil, Mexico, and Argentina.

**Table S5:** Characteristics of COVID-19 cases that required ICU admission.

Characteristic	Brazil	Mexico	Colombia	Argentina	Chile	Total
	(*n* = 443,359)	(*n* = 25,572)	-	(*n* = 31,236)	(*n* = 7868)	(*n* = 508,035)
**Sex, *n* (%)**						
Female	193,156 (43.6)	10,306 (40.3)	-	12,203 (39.1)	-	215,665 (43.1) ^c^
Male	250,165 (56.4)	15,266 (59.7)	-	18,849 (60.3)	-	284,280 (56.8) ^c^
Missing	38 (0.0)	0 (0)	-	184 (0.6)	-	222 (0) ^c^
**Age groups, *n* (%)**						
0–4 years	5500 (1.2)	554 (2.2)	-	190 (0.6)	-	6244 (1.2) ^c^
5–17 years	3115 (0.7)	371 (1.5)	-	267 (0.9)	-	3753 (0.8) c
18–29 years	14,232 (3.2)	1266 (5.0)	-	837 (2.7)	-	16,335 (3.3) ^c^
30–39 years	38,871 (8.8)	2420 (9.5)	-	1851 (5.9)	-	43,142 (8.6) ^c^
40–49 years	63,611 (14.3)	3723 (14.6)	-	3800 (12.2)	-	71,134 (14.2) ^c^
50–64 years	132,296 (29.8)	7674 (30.0)	-	9959 (31.9)	-	149,929 (30) ^c^
65–74 years	89,529 (20.2)	4867 (19.0)	-	7560 (24.2)	-	101,956 (20.4) ^c^
75–84 years	63,334 (14.3)	3297 (12.9)	-	4851 (15.5)	-	71,482 (14.3) ^c^
85+ years	32,639 (7.4)	1400 (5.5)	-	1914 (6.1)	-	35,953 (7.2) ^c^
Missing	232 (0.1)	0 (0)	-	7 (0.0)	-	239 (0) ^c^
3–5 years	-	-	-	-	47 (0.6)	47 (0.6) ^d^
6–11 years	-	-	-	-	57 (0.7)	57 (0.7) ^d^
12–20 years	-	-	-	-	112 (1.4)	112 (1.4) ^d^
21–30 years	-	-	-	-	275 (3.5)	275 (3.5) ^d^
31–40 years	-	-	-	-	550 (7.0)	550 (7.0) ^d^
41–50 years	-	-	-	-	756 (9.6)	756 (9.6) ^d^
51–60 years	-	-	-	-	1403 (17.8)	1403 (17.8) ^d^
61–70 years	-	-	-	-	2060 (26.2)	2060 (26.2) ^d^
71–80 years	-	-	-	-	1839 (23.4)	1839 (23.4) ^d^
80+ years	-	-	-	-	769 (9.8)	769 (9.8) ^d^
Missing	-	-	-	-	-	-
**Period, *n* (%)**						
2021Q1	139,585 (31.5)	1679 (6.6)	-	5312 (17.0)	-	146,576 (29.3) ^c^
2021Q2	123,116 (27.8)	745 (2.9)	-	15,863 (50.8)	-	139,724 (27.9) ^c^
2021Q3	66,109 (14.9)	4896 (19.1)	-	5037 (16.1)	-	76,042 (15.2) ^c^
2021Q4	49,873 (11.2)	2575 (10.1)	-	912 (2.9)	-	53,360 (10.7) ^c^
2022Q1	29,468 (6.6)	5634 (22.0)	-	3906 (12.5)	-	39,008 (7.8) ^c^
2022Q2	14,314 (3.2)	3456 (13.5)	-	206 (0.7)	-	17,976 (3.6) ^c^
2022Q3	9515 (2.1)	4970 (19.4)	-	-	-	-
2022Q4	11,379 (2.6)	1208 (4.7)	-	-	-	-
**Year, *n* (%)**						
2021	378,683 (85.4)	9895 (38.7)	-	26,961 (86.3)	-	415,539 (83.1) ^c^
2022	64,676 (14.6)	15,268 (59.7)	-	4091 (13.1)	-	84,035 (16.8) ^c^
Missing	0 (0)	409 (1.6)	-	184 (0.6)	-	593 (0.1) ^c^
**Comorbidities, *n*(%)**						
Chronical cardiac illness	124,518 (28.1)	1308 (5.1)	-	-	-	-
Hematologic disease	178,113 (40.2)	-	-	-	-	-
Carrier chromosomal disease immunological fragility	179,687 (40.5)	-	-	-	-	-
Hepatic disease	176,931 (39.9)	-	-	-	-	-
Diabetes	113,164 (25.5)	7946 (31.1)	-	-	-	-
Chronical neurological neuromuscular illness	168,840 (38.1)	-	-	-	-	-
Decompensated chronical respiratory diseases	180,399 (40.7)	-	-	-	-	-
Immunosuppression	172,012 (38.8)	657 (2.6)	-	-	-	-
Renal disease	167,002 (37.7)	1181 (4.6)	-	-	-	-
Obesity	141,536 (31.9)	5577 (21.8)	-	-	-	-
Pregnant	141,536 (31.9)	-	-	-	-	-
Neoplasia/Cancer	2974 (0.7)	-	-	-	-	-
COPD	-	756 (3.0)	-	-	-	-
Asthma	-	439 (1.7)	-	-	-	-
Hypertension	-	9216 (36.0)	-	-	-	-
Arthritis	-	-	-	-	-	-
Orphan diseases	-	-	-	-	-	-
HIV	-	-	-	-	-	-
Smoking	-	1570 (6.2)	-	-	-	-
Other comorbidities	89,296 (20.1)	797 (3.1)	-		-	-
**Vaccination status, *n* (%) ^a^**						
Yes	131,370 (29.6)	-	-	-	-	131,370 (29.6) ^b^
No	311,989 (70.4)	-	-	-	-	311,989 (70.4) ^b^

^a^ ‘Vaccination status = Yes’ when subject has received one or two doses of the initial COVID-19 vaccine (not including booster doses). ^b^ Percentages over the total number of cases in Brazil. ^c^ Percentages over the total number of cases excluding Chile and Colombia. ^d^ Percentages over the total number of cases in Chile.

**Table S6:** Characteristics of deceased COVID-19 cases per country.

Characteristic	Brazil	Mexico	Colombia	Argentina	Chile	Total
	(*n* = 424,606)	(*n* = 158,298)	(*n* = 94,354)	(*n* = 79,615)	(*n* = 13,068)	(*n* = 769,941)
**Sex, *n* (%)**						
Female	190,150 (44.8)	63,932 (40.4)	37,553 (39.8)	33,791 (42.4)	-	325,426 (43.0) ^b^
Male	234,421 (55.2)	94,366 (59.6)	56,780 (60.2)	45,198 (56.8)	-	430,765 (56.9) ^b^
Missing	35 (0.0)	0 (0)	21 (0.0)	626 (0.8)	-	682 (0.1) ^b^
**Age groups, *n* (%)**						
0–4 years	1010 (0.2)	417 (0.3)	120 (0.1)	80 (0.1)	-	1627 (0.2) ^b^
5–17 years	862 (0.2)	450 (0.3)	134 (0.1)	149 (0.2)	-	1595 (0.2) ^b^
18–29 years	6657 (1.6)	3069 (1.9)	1197 (1.3)	669 (0.8)	-	11,592 (1.5) ^b^
30–39 years	21,169 (5.0)	8412 (5.3)	3413 (3.6)	1973 (2.5)	-	34,967 (4.6) ^b^
40–49 years	42,696 (10.1)	16,974 (10.7)	7544 (8.0)	5229 (6.6)	-	72,443 (9.6) ^b^
50–64 years	117,595 (27.7)	47,866 (30.2)	24,450 (25.9)	18,224 (22.9)	-	208,135 (27.5) ^b^
65–74 years	99,950 (23.5)	39,167 (24.7)	22,231 (23.6)	19,935 (25.0)	-	181,283 (24) ^b^
75–84 years	82,269 (19.4)	28,925 (18.3)	20,781 (22.0)	19,269 (24.2)	-	151,244 (20) ^b^
85+ years	52,335 (12.3)	13,018 (8.2)	14,484 (15.4)	14,032 (17.6)	-	93,869 (12.4) ^b^
Missing	63 (0.0)	0 (0)	0 (0)	55 (0.1)	-	118 (0) ^b^
3–5 years	-	-	-	-	5 (0.0) ^c^	5 (0.0) ^d^
6–11 years	-	-	-	-	8 (0.1) ^c^	8 (0.1) ^d^
12–20 years	-	-	-	-	29 (0.2) ^c^	29 (0.2) ^d^
21–30 years	-	-	-	-	78 (0.6) ^c^	78 (0.6) ^d^
31–40 years	-	-	-	-	157 (1.2) ^c^	157 (1.2) ^d^
41–50 years	-	-	-	-	368 (2.8) ^c^	368 (2.8) ^d^
51–60 years	-	-	-	-	956 (7.3) ^c^	956 (7.3) ^d^
61–70 years	-	-	-	-	1850 (14.2) ^c^	1850 (14.2) ^d^
71–80 years	-	-	-	-	3288 (25.2) ^c^	3288 (25.2) ^d^
80+ years	-	-	-	-	6329 (48.4) ^c^	6329 (48.4) ^d^
Missing	-	-	-	-	-	-
**Period, *n* (%)**						
2021Q1	147,279 (34.7)	13,853 (8.8)	19,530 (20.7)	13,385 (16.8)	-	194,047 (25.6) ^b^
2021Q2	112,694 (26.5)	3265 (2.1)	47,372 (50.2)	42,449 (53.3)	-	205,780 (27.2) ^b^
2021Q3	57,987 (13.7)	36,071 (22.8)	11,988 (12.7)	11,337 (14.2)	-	117,383 (15.5) ^b^
2021Q4	46,532 (11.0)	23,572 (14.9)	3618 (3.8)	1620 (2.0)	-	73,884 (9.8) ^b^
2022Q1	29,602 (7.0)	35,270 (22.3)	9203 (9.8)	10,558 (13.3)	-	52,890 (7) ^b^
2022Q2	12,163 (2.9)	14,536 (9.2)	690 (0.7)	266 (0.3)	-	27,655 (3.7) ^b^
2022Q3	8547 (2.0)	23,305 (14.7)	1359 (1.4)	-	-	-
2022Q4	9802 (2.3)	6407 (4.0)	594 (0.6)	-	-	-
**Year, *n* (%)**						
2021	364,492 (85.8)	76,761 (48.5)	82,508 (87.4)	68,219 (85.7)	-	591,980 (78.2) ^b^
2022	60,114 (14.2)	79,518 (50.2)	11,846 (12.6)	10,770 (13.5)	-	152,555 (20.2) ^b^
Missing	0 (0)	2019 (1.3)	0 (0)	626 (0.8)	-	2645 (0.3) ^b^
**Comorbidities, *n*(%)**						
Chronical cardiac illness	121,253 (28.6)	7824 (4.9)	-	-	-	-
Hematologic disease	176,726 (41.6)	-	-	-	-	-
Carrier chromosomal disease immunological fragility	178,586 (42.1)	-	-	-	-	-
Hepatic disease	175,184 (41.3)		-	-	-	-
Diabetes	109,293 (25.7)	56,506 (35.7)	17,008 (18.0)	-	-	-
Chronical neurological neuromuscular illness	164,479 (38.7)	-	-	-	-	-
Decompensated chronical respiratory diseases	179,204 (42.2)	-	-	-	-	-
Immunosuppression	169,337 (39.9)	3705 (2.3)	-	-	-	-
Renal disease	164,414 (38.7)	11,904 (7.5)	-	-	-	-
Obesity	146,976 (34.6)	29,914 (18.9)	-	-	-	-
Pregnant	1103 (0.7)	-	-	-	-	-
Neoplasia/Cancer	4562 (1.1)	-	2947 (3.1)	-	-	-
COPD	-	6758 (4.3)	-	-	-	-
Asthma	-	2561 (1.6)	-	-	-	-
Hypertension	-	68,989 (43.6)	38,728 (41.0)	-	-	-
Arthritis	-	-	886 (0.9)	-	-	-
Orphan diseases	-	-	465 (0.5)	-	-	-
HIV	-	-	337 (0.4)	-	-	-
Smoking	-	11,100 (7.0)	-	-	-	-
Other comorbidities	89,408 (21.1)	8130 (5.1)	-		-	-
**Vaccination status, *n* (%) ^a^**						
Yes	122,873 (28.9)	-	29,333 (31.1)	-	-	152,206 (29.3) ^b^
No	301,733 (71.1)	-	65,021 (68.9)	-	-	366,754 (70.7) ^b^

^a^ ‘Vaccination status = Yes’ when subject has received one or two doses of the initial COVID-19 vaccine (not including booster doses) ^b^ Percentages over the total number of cases excluding Chile ^c^ Data from week 40 of 2021 to week 51 of 2022 ^d^ Percentages over the total number of cases in Chile ^e^ Percentages over the total number of cases in Brazil and Colombia.

**Figure S2:**
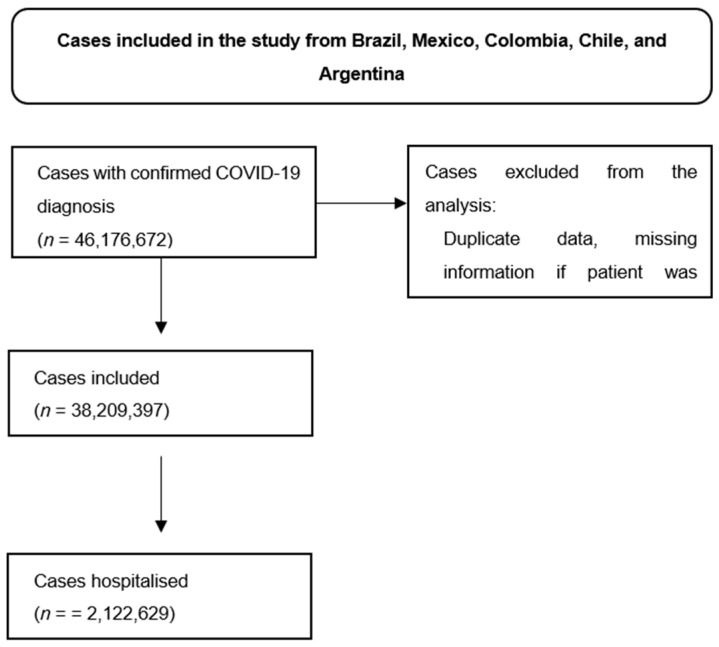
Patient selection flow chart including all countries.

**Figure S3:**
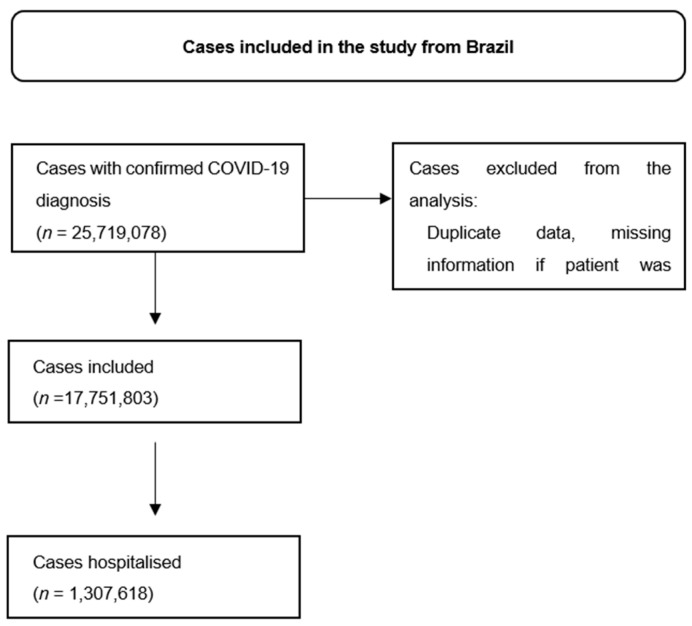
Patient selection in Brazil’s database.

**Figure S4:**
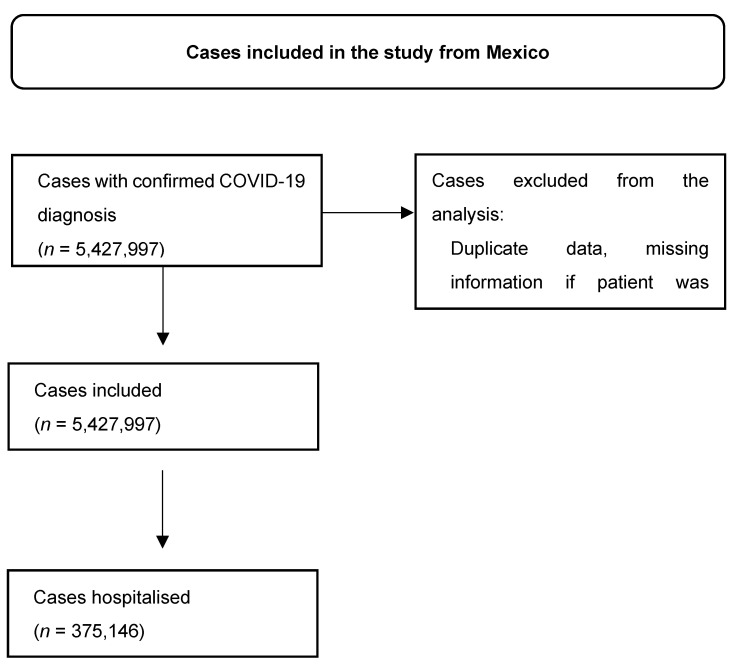
Patient selection in Mexico’s database.

**Figure S5:**
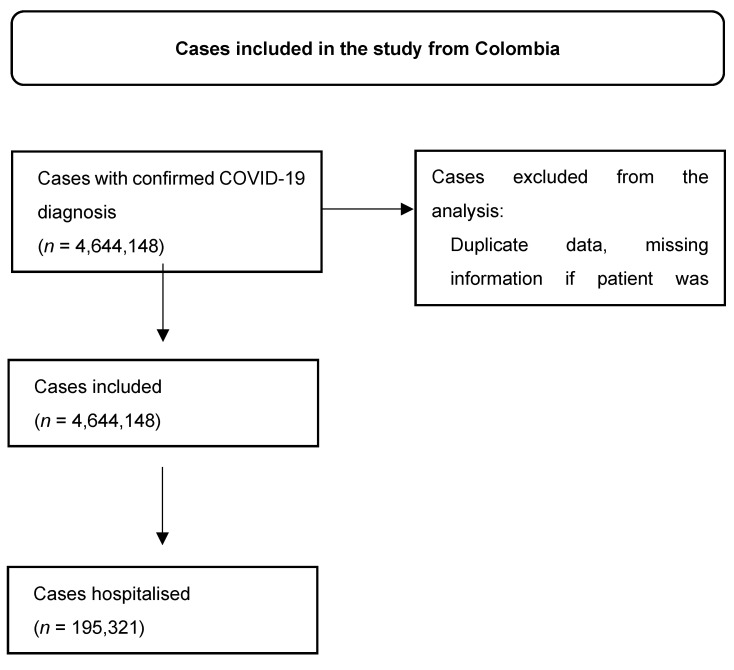
Patient selection in Colombia’s database.

**Figure S6:**
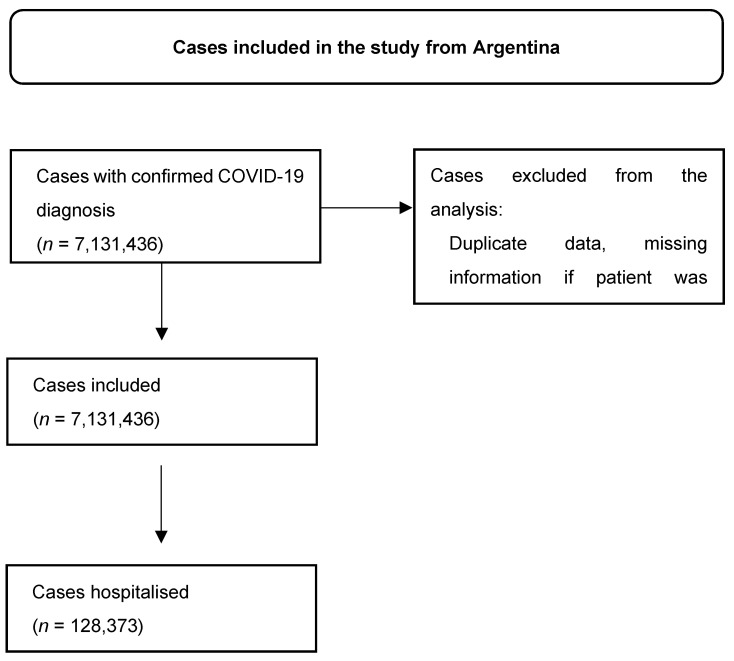
Patient selection in Argentina’s database.

**Figure S7:**
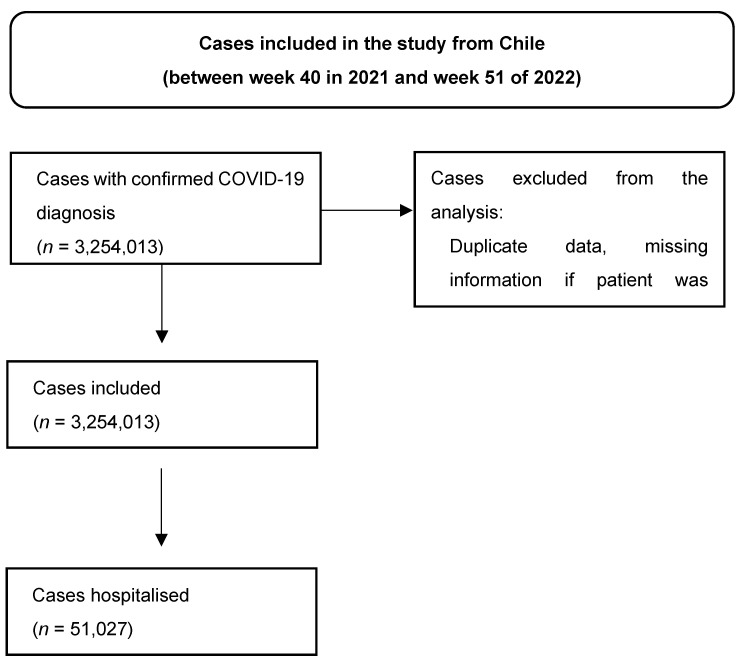
Patient selection in Chile’s database.

**Figure S8:**
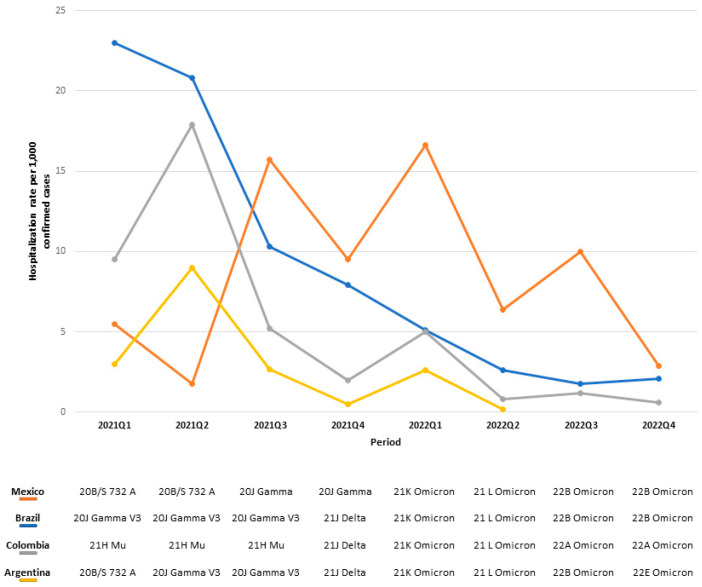
Hospitalization rate per 1000 confirmed COVID-19 cases per country, indicating the predominant SARS-CoV-2 by period.

**Figure S9:**
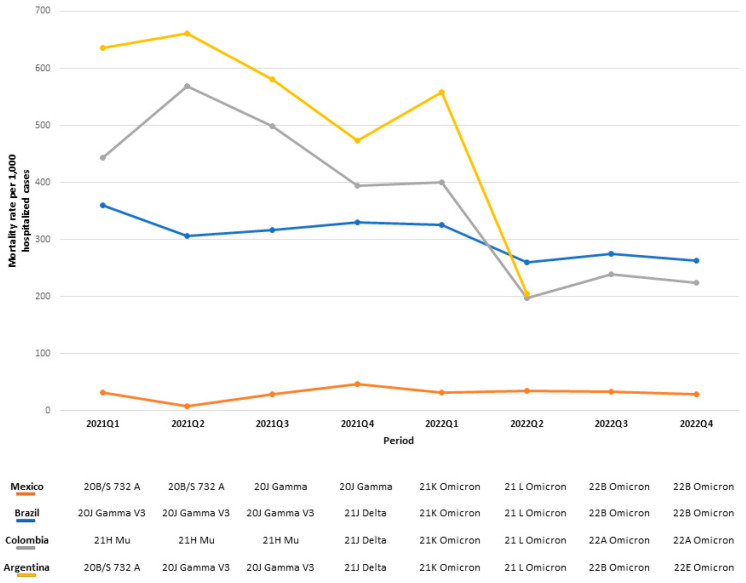
Mortality rate per 1000 hospitalized COVID-19 cases per country, indicating the predominant SARS-CoV-2 by period.

The authors state that the scientific conclusions are unaffected. This correction was approved by the Academic Editor. The original publication has also been updated.
