# Supplementary material for: Severe COVID-19 Outcomes in Five Latin American Countries in the Postvaccination Era
Source: Viruses. 2024 Jun 26;16(7):1025. doi: 10.3390/v16071025 (PMC11281361; doi:10.3390/v16071025)
Supplement: Supplementary file 1 [file viruses-16-01025-s001.zip › viruses-3022721-supplementary.pdf]

Table S1. Procedure codes for ventilatory support used in Colombia.

Code	Procedure
**9390**	**Continuous positive pressure breathing**
939000	Continuous positive pressure breathing sod
**9391**	**Intermittent positive pressure breathing**
939100	Intermittent positive pressure breath sod
**9601**	**Nasopharyngeal airway insertion**
960100	Sod nasopharyngeal airway insertion
**9602**	**Oropharyngeal airway insertion**
960200	Oropharyngeal airway insertion sod
**9603**	**Insertion of obturated esophageal airway**
960300	Sod esophageal obturated airway insertion
**9604**	**Endotracheal tube insertion**
960401	Endotracheal tube insertion with retrograde technique
960402	Double lamp endotracheal tube insertion
960403	Endotracheal tube insertion with selective blocker
960404	Insertion of endotracheal tube with luminous probe
960405	Transtracheal jet cannula insertion
960406	Insertion of endotracheal tube under endoscopic vision
**9605**	**Other respiratory tract intubation**

Table S2. Predominant SARS-CoV-2 variants per country.

Q	**Mexico**		**Brazil**		**Chile**		**Colombia**		**Argentina**	
	2021	2022	2021	2022	2021	2022	2021	2022	2021	2022
Q1	20B/S 732 A	21K Omicron	20J Gamma V3	21K Omicron	21G Lambda	21K Omicron	21H Mu	21K Omicron	20B/S 732 A	21K Omicron
Q2	20B/S 732 A	21 L Omicron	20J Gamma V3	21 L Omicron	20J Gamma	21 L Omicron	21H Mu	21 L Omicron	20J Gamma V3	21 L Omicron
Q3	20J Gamma	22B Omicron	20J Gamma V3	22B Omicron	20J Gamma	22B Omicron	21H Mu	22A Omicron	20J Gamma V3	22B Omicron
Q4	20J Gamma	22B Omicron	21J Delta	22B Omicron	21J Delta	22E Omicron	21J Delta	22A Omicron	21J Delta	22E Omicron

Adapted from Hodcrof E. Overview of Variants in Countries. CoVariants [Internet]. 2023. Available Online: https://covariants.org/per-country (Accessed on: 10 January 2023).

Table S3. Characteristics of hospitalized COVID-19 cases per country.

**Characteristic**	**Brazil (*n* = 1,307,618)**	**Mexico (*n* = 375,146)**	**Colombia (*n* = 195,321)**	**Argentina (*n* = 128,373)**	**Chile (*n* = 116,171)**	**Total (*n* = 2,122,629)**
**Sex, *n* (%)**						
Female	589,363 (45.1)	166,951 (44.5)	83,383 (42.7)	56,582 (44.1)	57,028 (49.1)	953,307 (44.9)
Male	718,146 (54.9)	208,195 (55.5)	111,687 (57.2)	70,914 (55.2)	59,143 (50.9)	1,168,085 (55)
Missing	109 (0.0)	0 (0)	251 (0.1)	877 (0.7)	0 (0)	1237 (0.1)
**Age groups, *n* (%)**						
0–4 years	22,043 (1.7)	7601 (2.0)	8172 (4.2)	1847 (1.4)	-	39,663 (2) [b]
5–17 years	12,172 (0.9)	8064 (2.1)	3421 (1.8)	2709 (2.1)	-	26,366 (1.3) [b]
18–29 years	55,755 (4.3)	23,659 (6.3)	8750 (4.5)	5689 (4.4)	-	93,853 (4.7) [b]
30–39 years	138,140 (10.6)	36,521 (9.7)	14,413 (7.4)	9535 (7.4)	-	198,609 (9.9) [b]
40–49 years	205,970 (15.8)	50,898 (13.6)	21,686 (11.1)	15,545 (12.1)	-	294,099 (14.7) [b]
50–64 years	379,244 (29.0)	105,794 (28.2)	52,613 (26.9)	33,800 (26.3)	-	571,451 (28.5) [b]
65–74 years	230,153 (17.6)	71,198 (19.0)	37,507 (19.2)	25,098 (19.6)	-	363,956 (18.1) [b]
75–84 years	167,580 (12.8)	49,594 (13.2)	30,902 (15.8)	20,887 (16.3)	-	268,963 (13.4) [b]
85+ years	95,981 (7.3)	21,817 (5.8)	17,853 (9.1)	13,197 (10.3)	-	148,848 (7.4) [b]
Missing	580 (0.0)	0 (0)	4 (0.0)	66 (0.1)	-	650 (0) [b]
3–5 years	-	-	-	-	415 (0.8) [c]	415 (0.8) [d]

6–11 years	-	-	-	-	618 (1.2) [c]	618 (1.2) [d]
12–20 years	-	-	-	-	1698 (3.3) [c]	1698 (3.3) [d]
21–30 years	-	-	-	-	3914 (7.7) [c]	3914 (7.7) [d]
31–40 years	-	-	-	-	4445 (8.7) [c]	4445 (8.7) [d]
41–50 years	-	-	-	-	4108 (8.1) [c]	4108 (8.1) [d]
51–60 years	-	-	-	-	6394 (12.5) [c]	6394 (12.5) [d]
61–70 years	-	-	-	-	8993 (17.6) [c]	8993 (17.6) [d]
71–80 years	-	-	-	-	10,313 (20.2) [c]	10,313 (20.2) [d]
80+ years	-	-	-	-	10,129 (19.9) [c]	10,129 (19.9) [d]
Missing	-	-	-	-	65,144	-
**Period, *n* (%)**						
2021Q1	408,898 (31.3)	29,653 (7.9)	43,982 (22.5)	21,071 (16.4)	-	-
2021Q2	368,765 (28.2)	9874 (2.6)	83,281 (42.6)	64,180 (50.0)	-	-
2021Q3	183,413 (14.0)	85,331 (22.7)	24,070 (12.3)	19,510 (15.20)	-	-
2021Q4	140,706 (10.8)	51,688 (13.8)	9179 (4.7)	3428 (2.70)	-	-
2022Q1	90,900 (7.0)	89,848 (24.0)	23,007 (11.8)	18,889 (14.70)	-	-
2022Q2	46,659 (3.6)	34,745 (9.3)	3485 (1.8)	1295 (1.0)	-	-
2022Q3	31,101 (2.4)	54,065 (14.4)	5671 (2.9)	-	-	-
2022Q4	37,176 (2.8)	15,770 (4.2)	2646 (1.4)	-	-	-
**Year, *n* (%)**						
2021	1,101,782 (84.3)	176,546 (47.1)	160,512 (82.2)	107,413 (83.7)	48,058 (41.4)	1,594,311 (75.1)
2022	205,836 (15.7)	194,428 (51.8)	34,809 (17.8)	20,083 (15.6)	68,113 (58.6)	523,269 (24.7)
Missing	0 (0)	4172 (1.1)	0 (0)	877 (0.7)	0 (0)	5049 (0.2)
**Comorbidities, *n* (%)**						
Chronical cardiac illness	321,316 (24.6)	16,181 (4.3)	-	-	-	-
Hematologic disease	459,122 (35.1)	-	-	-	-	-
Carrier chromosomal disease immunological fragility	463,022 (35.4)	-	-	-	-	-
Hepatic disease	456,462 (34.9)		-	-	-	-
Diabetes	300,120 (23.0)	110,877 (29.6)	27,168 (13.9)	-	-	-
Chronical neurological neuromuscular illness	434,150 (33.2)	-	-	-	-	-
Decompensated chronical respiratory diseases	464,673 (35.5)	-	-	-	-	-
Immunosuppression	443,472 (33.9)	8535 (2.3)	-	-	-	-
Renal disease	436,226 (33.4)	22,630 (6.0)	-	-	-	-
Obesity	383,522 (29.3)	62,268 (16.6)	-	-	-	-
Pregnant	12,775 (2.6)	-	-	-	-	-
Neoplasia/Cancer	7708 (0.6)	-	4900 (2.5)	-	-	-
COPD	-	13,088 (3.5)	-	-	-	-
Asthma	-	6970 (1.9)	-	-	-	-
Hypertension	-	132,337 (35.3)	62,994 (32.3)	-	-	-
Arthritis	-	-	1461 (0.7)	-	-	-
Orphan diseases	-	-	1194 (0.6)	-	-	-
HIV	-	-	739 (0.4)	-	-	-
Smoking	-	24,638 (6.6)	-	-	-	-
Other comorbidities	239,059 (18.3)	17,846 (4.8)	-	-	-	-
**Vaccination status, *n* (%)** [a]						
Yes	386,288 (29.5)	-	112,147 (57.4)	-	-	498,435 (33.2)
No	921,330 (70.5)	-	83,174 (42.6)	-	-	1,004,504 (66.8)

[a] 'Vaccination status = Yes' when subject has received one or two doses of the initial COVID-19 vaccine (not including booster doses). [b] Percentages over the total number of cases excluding Chile. [c] Data from week 40 of 2021 to week 51 of 2022. [d] Percentages over the total number of cases in Chile.

Table S4. Characteristics of COVID-19 cases requiring ventilatory support per country.

Characteristic	Brazil	Mexico	Colombia	Argentina	Chile	Total
	(*n* = 938,850)	(*n* = 33,612)	(*n* = 1515)	(*n* = 18,728)	-	(*n* = 992,705)
**Sex, *n* (%)**						
Female	418,215 (44.5)	13,094 (39.0)	641 (42.3)	6991 (37.3)	-	438,941 (44.2)
Male	520,582 (55.4)	20,518 (61.0)	874 (57.7)	11,642 (62.2)	-	553,616 (55.8)
Missing	53 (0.0)	0 (0)	0 (0)	95 (0.5)	-	148 (0)
**Age groups, *n* (%)**						
0–4 years	10,258 (1.1)	424 (1.3)	17 (1.1)	80 (0.4)	-	10,779 (1.1) [b]
5–17 years	5666 (0.6)	326 (1.0)	2 (0.1)	117 (0.6)	-	6111 (0.6) [b]
18–29 years	33,986 (3.6)	1185 (3.5)	37 (2.4)	416 (2.2)	-	35,624 (3.6) [b]
30–39 years	95,118 (10.1)	2679 (8.0)	101 (6.7)	1021 (5.5)	-	98,919 (10) [b]
40–49 years	148,808 (15.9)	4766 (14.2)	228 (15.0)	2355 (12.6)	-	156,157 (15.7) [b]
50–64 years	281,941 (30.0)	10,961 (32.6)	553 (36.5)	6506 (34.7)	-	299,961 (30.2) [b]
65–74 years	171,281 (18.2)	7384 (22.0)	328 (21.7)	4786 (25.6)	-	183,779 (18.5) [b]
75–84 years	122,414 (13.0)	4478 (13.3)	208 (13.7)	2700 (14.4)	-	129800 (13.1) [b]
85+ years	69,086 (7.4)	1409 (4.2)	41 (2.7)	745 (4.0)	-	71,281 (7.2) [b]
Missing	292 (0.0)	0 (0)	0 (0)	2 (0.0)	-	294 (0) [b]
**Period, *n* (%)**						
2021Q1	304,068 (32.4)	3560 (10.6)	-	2989 (16.0)	-	310,617 (31.3) [d]
2021Q2	276,631 (29.5)	1005 (3.0)	-	10,258 (54.8)	-	287,894 (29) [d]
2021Q3	135,467 (14.4)	7437 (22.1)	-	2950 (15.8)	-	145,854 (14.7) [d]
2021Q4	103,192 (11.0)	5742 (17.1)	-	473 (2.5)	-	109,407 (11) [d]
2022Q1	54,470 (5.8)	6475 (19.3)	-	1985 (10.6)	-	62,930 (6.3) [d]
2022Q2	26,517 (2.8)	3051 (9.1)	-	73 (0.4)	-	29,641 (3) [d]
2022Q3	17,916 (1.9)	5051 (15.0)	-	-	-	22,967 (2.3) [d]
2022Q4	20,589 (2.2)	709 (2.1)	-	-	-	21,298 (2.1) [d]
**Year, *n* (%)**						
2021	819,358 (87.3)	17,744 (52.8)	-	16,580 (88.5)	-	853,682 (86.1)
2022	119,492 (12.7)	15,286 (45.5)	-	2053 (11.0)	-	136,831 (13.8)
Missing	0 (0)	582 (1.7)	-	95 (0.5)	-	9062 (0.9)
**Comorbidities, *n* (%)**						
Chronical cardiac illness	167,217 (27.8)	1679 (5.0)	-	-	-	-
Hematologic disease	250,408 (41.6)	-	-	-	-	-
Carrier chromosomal disease immunological fragility	252,632 (42.0)	-	-	-	-	-
Hepatic disease	249,883 (41.6)		-	-	-	-
Diabetes	163,988 (27.3)	11,134 (33.1)	-	-	-	-
Chronical neurological neuromuscular illness	232,792 (38.7)	-	-	-	-	-
Decompensated chronical respiratory diseases	252,649 (42.0)	-	-	-	-	-
Immunosuppression	241,364 (40.2)	861 (2.6)	-	-	-	-
Renal disease	239,695 (39.9)	1894 (5.6)	-	-	-	-
Obesity	209,745 (34.9)	8194 (24.4)	-	-	-	-
Pregnant	7503 (1.6)	-	-	-	-	-
Neoplasia /Cancer	4012 (0.7)	-	-	-	-	-
COPD	-	1113 (3.3)	-	-	-	-
Asthma	-	655 (1.9)	-	-	-	-
Hypertension	-	13,370 (39.8)	-	-	-	-
Arthritis	-	-	-	-	-	-
Orphan diseases	-	-	-	-	-	-

HIV	-	-	-	-	-	-
Smoking	-	2453 (7.3)	-	-	-	-
Other comorbidities	128,470 (21.4)	1653 (4.9)	-		-	-
**Vaccination status, *n* (%)** [a]						
Yes	269,084 (28.7) [c]	-	-	-	-	269,084 (28.7) [c]
No	669,766 (71.3) [c]	-	-	-	-	669,766 (71.3) [c]

[a] 'Vaccination status = Yes' when subject has received one or two doses of the initial COVID-19 vaccine (not including booster doses). [b] Percentages over the total number of cases excluding Chile. [c] Percentages over the total number of cases in Brazil. [d] Percentages over the total number of cases in Brazil, Mexico, and Argentina.

Table S5. Characteristics of COVID-19 cases that required ICU admission.

Characteristic	Brazil	Mexico	Colombia	Argentina	Chile	Total
	(*n* = 443,359)	(*n* = 25,572)	-	(*n* = 31,236)	(*n* = 7868)	(*n* = 508,035)
**Sex, *n* (%)**						
Female	193,156 (43.6)	10,306 (40.3)	-	12,203 (39.1)	-	215,665 (43.1) [c]
Male	250,165 (56.4)	15,266 (59.7)	-	18,849 (60.3)	-	284,280 (56.8) [c]
Missing	38 (0.0)	0 (0)	-	184 (0.6)	-	222 (0) [c]
**Age groups, *n* (%)**						
0–4 years	5500 (1.2)	554 (2.2)	-	190 (0.6)	-	6244 (1.2) [c]
5–17 years	3115 (0.7)	371 (1.5)	-	267 (0.9)	-	3753 (0.8) c
18–29 years	14,232 (3.2)	1266 (5.0)	-	837 (2.7)	-	16,335 (3.3) [c]
30–39 years	38,871 (8.8)	2420 (9.5)	-	1851 (5.9)	-	43,142 (8.6) [c]
40–49 years	63,611 (14.3)	3723 (14.6)	-	3800 (12.2)	-	71,134 (14.2) [c]
50–64 years	132,296 (29.8)	7674 (30.0)	-	9959 (31.9)	-	149,929 (30) [c]
65–74 years	89,529 (20.2)	4867 (19.0)	-	7560 (24.2)	-	101,956 (20.4) [c]
75–84 years	63,334 (14.3)	3297 (12.9)	-	4851 (15.5)	-	71,482 (14.3) [c]
85+ years	32,639 (7.4)	1400 (5.5)	-	1914 (6.1)	-	35,953 (7.2) [c]
Missing	232 (0.1)	0 (0)	-	7 (0.0)	-	239 (0) [c]
3–5 years	-	-	-	-	47 (0.6)	47 (0.6) [d]
6–11 years	-	-	-	-	57 (0.7)	57 (0.7) [d]
12–20 years	-	-	-	-	112 (1.4)	112 (1.4) [d]
21–30 years	-	-	-	-	275 (3.5)	275 (3.5) [d]
31–40 years	-	-	-	-	550 (7.0)	550 (7.0) [d]
41–50 years	-	-	-	-	756 (9.6)	756 (9.6) [d]
51–60 years	-	-	-	-	1403 (17.8)	1403 (17.8) [d]
61–70 years	-	-	-	-	2060 (26.2)	2060 (26.2) [d]
71–80 years	-	-	-	-	1839 (23.4)	1839 (23.4) [d]
80+ years	-	-	-	-	769 (9.8)	769 (9.8) [d]
Missing	-	-	-	-	-	-
**Period, *n* (%)**						
2021Q1	139,585 (31.5)	1679 (6.6)	-	5312 (17.0)	-	146,576 (29.3) [c]
2021Q2	123,116 (27.8)	745 (2.9)	-	15,863 (50.8)	-	139,724 (27.9) [c]
2021Q3	66,109 (14.9)	4896 (19.1)	-	5037 (16.1)	-	76,042 (15.2) [c]
2021Q4	49,873 (11.2)	2575 (10.1)	-	912 (2.9)	-	53,360 (10.7) [c]
2022Q1	29,468 (6.6)	5634 (22.0)	-	3906 (12.5)	-	39,008 (7.8) [c]
2022Q2	14,314 (3.2)	3456 (13.5)	-	206 (0.7)	-	17,976 (3.6) [c]
2022Q3	9515 (2.1)	4970 (19.4)	-	-	-	-
2022Q4	11,379 (2.6)	1208 (4.7)	-	-	-	-
**Year, *n* (%)**						
2021	378,683 (85.4)	9895 (38.7)	-	26,961 (86.3)	-	415,539 (83.1) [c]
2022	64,676 (14.6)	15,268 (59.7)	-	4091 (13.1)	-	84,035 (16.8) [c]
Missing	0 (0)	409 (1.6)	-	184 (0.6)	-	593 (0.1) [c]
**Comorbidities, *n*(%)**						
Chronical cardiac illness	124,518 (28.1)	1308 (5.1)	-	-	-	-

Hematologic disease	178,113 (40.2)	-	-	-	-	-
Carrier chromosomal disease immunological fragility	179,687 (40.5)	-	-	-	-	-
Hepatic disease	176,931 (39.9)	-	-	-	-	-
Diabetes	113,164 (25.5)	7946 (31.1)	-	-	-	-
Chronical neurological neuromuscular illness	168,840 (38.1)	-	-	-	-	-
Decompensated chronical respiratory diseases	180,399 (40.7)	-	-	-	-	-
Immunosuppression	172,012 (38.8)	657 (2.6)	-	-	-	-
Renal disease	167,002 (37.7)	1181 (4.6)	-	-	-	-
Obesity	141,536 (31.9)	5577 (21.8)	-	-	-	-
Pregnant	141,536 (31.9)	-	-	-	-	-
Neoplasia/Cancer	2974 (0.7)	-	-	-	-	-
COPD	-	756 (3.0)	-	-	-	-
Asthma	-	439 (1.7)	-	-	-	-
Hypertension	-	9216 (36.0)	-	-	-	-
Arthritis	-	-	-	-	-	-
Orphan diseases	-	-	-	-	-	-
HIV	-	-	-	-	-	-
Smoking	-	1570 (6.2)	-	-	-	-
Other comorbidities	89,296 (20.1)	797 (3.1)	-		-	-
**Vaccination status, *n* (%)** [a]						
Yes	131,370 (29.6)	-	-	-	-	131,370 (29.6) [b]
No	311,989 (70.4)	-	-	-	-	311,989 (70.4) [b]

[a] 'Vaccination status = Yes' when subject has received one or two doses of the initial COVID-19 vaccine (not including booster doses). [b] Percentages over the total number of cases in Brazil. [c] Percentages over the total number of cases excluding Chile and Colombia. [d] Percentages over the total number of cases in Chile.

Table S6. Characteristics of deceased COVID-19 cases per country.

Characteristic	Brazil	Mexico	Colombia	Argentina	Chile	Total
	**(*n* = 424,606)**	**(*n* = 158,298)**	**(*n* = 94,354)**	**(*n* = 79,615)**	**(*n* = 13,068)**	**(*n* = 769,941)**
**Sex, *n* (%)**						
Female	190,150 (44.8)	63,932 (40.4)	37,553 (39.8)	33,791 (42.4)	-	325,426 (43.0) [b]
Male	234,421 (55.2)	94,366 (59.6)	56,780 (60.2)	45,198 (56.8)	-	430,765 (56.9) [b]
Missing	35 (0.0)	0 (0)	21 (0.0)	626 (0.8)	-	682 (0.1) [b]
**Age groups, *n* (%)**						
0–4 years	1010 (0.2)	417 (0.3)	120 (0.1)	80 (0.1)	-	1627 (0.2) [b]
5–17 years	862 (0.2)	450 (0.3)	134 (0.1)	149 (0.2)	-	1595 (0.2) [b]
18–29 years	6657 (1.6)	3069 (1.9)	1197 (1.3)	669 (0.8)	-	11,592 (1.5) [b]
30–39 years	21,169 (5.0)	8412 (5.3)	3413 (3.6)	1973 (2.5)	-	34,967 (4.6) [b]
40–49 years	42,696 (10.1)	16,974 (10.7)	7544 (8.0)	5229 (6.6)	-	72,443 (9.6) [b]
50–64 years	117,595 (27.7)	47,866 (30.2)	24,450 (25.9)	18,224 (22.9)	-	208,135 (27.5) [b]
65–74 years	99,950 (23.5)	39,167 (24.7)	22,231 (23.6)	19,935 (25.0)	-	181,283 (24) [b]
75–84 years	82,269 (19.4)	28,925 (18.3)	20,781 (22.0)	19,269 (24.2)	-	151,244 (20) [b]
85+ years	52,335 (12.3)	13,018 (8.2)	14,484 (15.4)	14,032 (17.6)	-	93,869 (12.4) [b]
Missing	63 (0.0)	0 (0)	0 (0)	55 (0.1)	-	118 (0) [b]
3–5 years	-	-	-	-	5 (0.0) [c]	5 (0.0) [d]
6–11 years	-	-	-	-	8 (0.1) [c]	8 (0.1) [d]
12–20 years	-	-	-	-	29 (0.2) [c]	29 (0.2) [d]
21–30 years	-	-	-	-	78 (0.6) [c]	78 (0.6) [d]
31–40 years	-	-	-	-	157 (1.2) [c]	157 (1.2) [d]
41–50 years	-	-	-	-	368 (2.8) [c]	368 (2.8) [d]
51–60 years	-	-	-	-	956 (7.3) [c]	956 (7.3) [d]
61–70 years	-	-	-	-	1850 (14.2) [c]	1850 (14.2) [d]
71–80 years	-	-	-	-	3288 (25.2) [c]	3288 (25.2) [d]
80+ years	-	-	-	-	6329 (48.4) [c]	6329 (48.4) [d]
Missing	-	-	-	-	-	-
**Period, *n* (%)**						
2021Q1	147,279 (34.7)	13,853 (8.8)	19,530 (20.7)	13,385 (16.8)	-	194,047 (25.6) [b]
2021Q2	112,694 (26.5)	3265 (2.1)	47,372 (50.2)	42,449 (53.3)	-	205,780 (27.2) [b]
2021Q3	57,987 (13.7)	36,071 (22.8)	11,988 (12.7)	11,337 (14.2)	-	117,383 (15.5) [b]
2021Q4	46,532 (11.0)	23,572 (14.9)	3618 (3.8)	1620 (2.0)	-	73,884 (9.8) [b]
2022Q1	29,602 (7.0)	35,270 (22.3)	9203 (9.8)	10,558 (13.3)	-	52,890 (7) [b]
2022Q2	12,163 (2.9)	14,536 (9.2)	690 (0.7)	266 (0.3)	-	27,655 (3.7) [b]
2022Q3	8547 (2.0)	23,305 (14.7)	1359 (1.4)	-	-	-
2022Q4	9802 (2.3)	6407 (4.0)	594 (0.6)	-	-	-
**Year, *n* (%)**						
2021	364,492 (85.8)	76,761 (48.5)	82,508 (87.4)	68,219 (85.7)	-	591,980 (78.2) [b]
2022	60,114 (14.2)	79,518 (50.2)	11,846 (12.6)	10,770 (13.5)	-	152,555 (20.2) [b]
Missing	0 (0)	2019 (1.3)	0 (0)	626 (0.8)	-	2645 (0.3) [b]
**Comorbidities, *n*(%)**						
Chronical cardiac illness	121,253 (28.6)	7824 (4.9)	-	-	-	-

Hematologic disease	176,726 (41.6)	-	-	-	-	-
Carrier chromosomal disease immunological fragility	178,586 (42.1)	-	-	-	-	-
Hepatic disease	175,184 (41.3)		-	-	-	-
Diabetes	109,293 (25.7)	56,506 (35.7)	17,008 (18.0)	-	-	-
Chronical neurological neuromuscular illness	164,479 (38.7)	-	-	-	-	-
Decompensated chronical respiratory diseases	179,204 (42.2)	-	-	-	-	-
Immunosuppression	169,337 (39.9)	3705 (2.3)	-	-	-	-
Renal disease	164,414 (38.7)	11,904 (7.5)	-	-	-	-
Obesity	146,976 (34.6)	29,914 (18.9)	-	-	-	-
Pregnant	1103 (0.7)	-	-	-	-	-
Neoplasia/Cancer	4562 (1.1)	-	2947 (3.1)	-	-	-
COPD	-	6758 (4.3)	-	-	-	-
Asthma	-	2561 (1.6)	-	-	-	-
Hypertension	-	68,989 (43.6)	38,728 (41.0)	-	-	-
Arthritis	-	-	886 (0.9)	-	-	-
Orphan diseases	-	-	465 (0.5)	-	-	-
HIV	-	-	337 (0.4)	-	-	-
Smoking	-	11,100 (7.0)	-	-	-	-
Other comorbidities	89,408 (21.1)	8130 (5.1)	-		-	-
**Vaccination status, *n* (%)** [a]						
Yes	122,873 (28.9)	-	29,333 (31.1)	-	-	152,206 (29.3) [b]
No	301,733 (71.1)	-	65,021 (68.9)	-	-	366,754 (70.7) [b]

[a] 'Vaccination status = Yes' when subject has received one or two doses of the initial COVID-19 vaccine (not including booster doses) [b] Percentages over the total number of cases excluding Chile [c] Data from week 40 of 2021 to week 51 of 2022 [d] Percentages over the total number of cases in Chile [e] Percentages over the total number of cases in Brazil and Colombia.

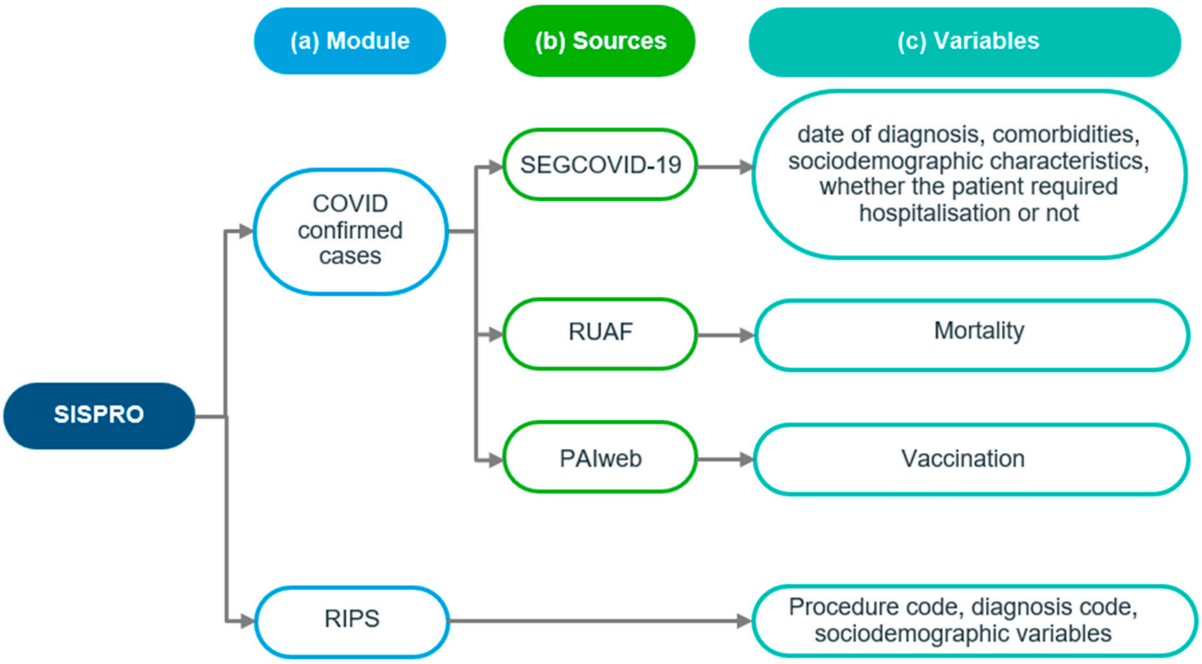

Figure S1. Databases in Colombia.

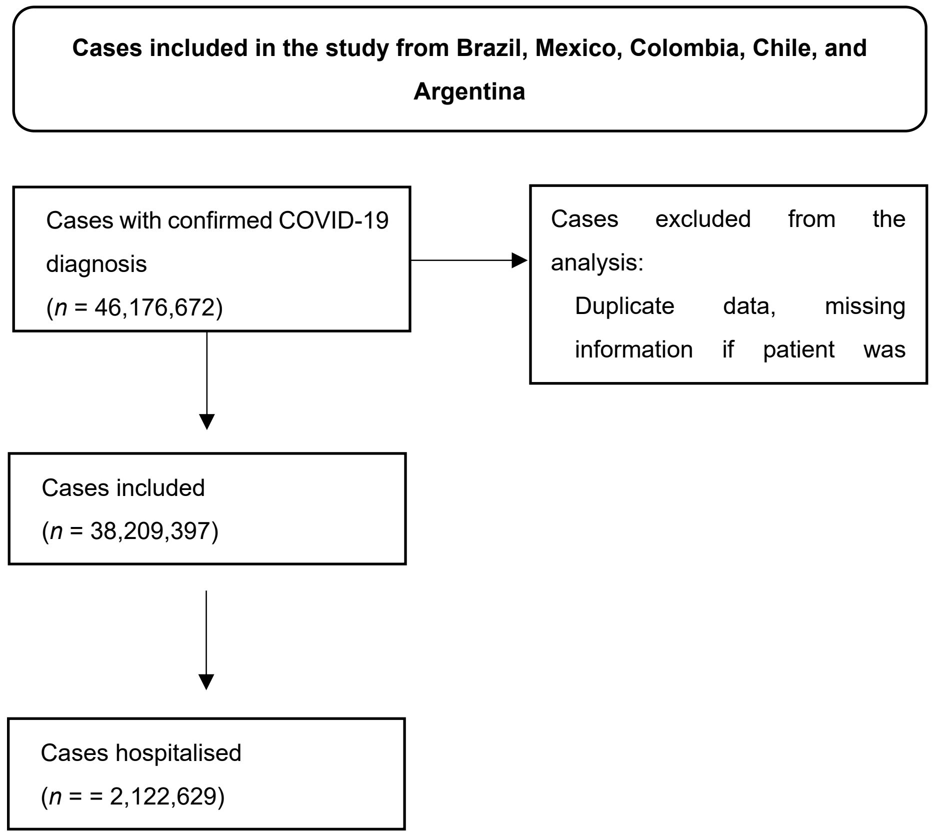

Figure S2. Patient selection flow chart including all countries.

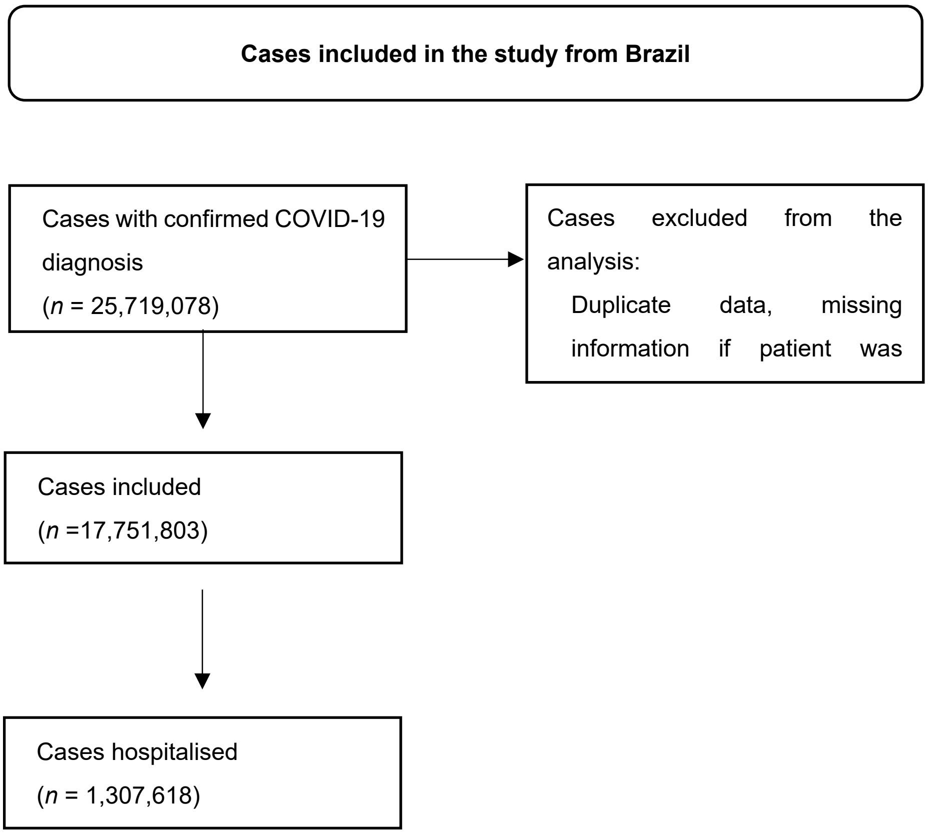

Figure S3. Patient selection in Brazil's database.

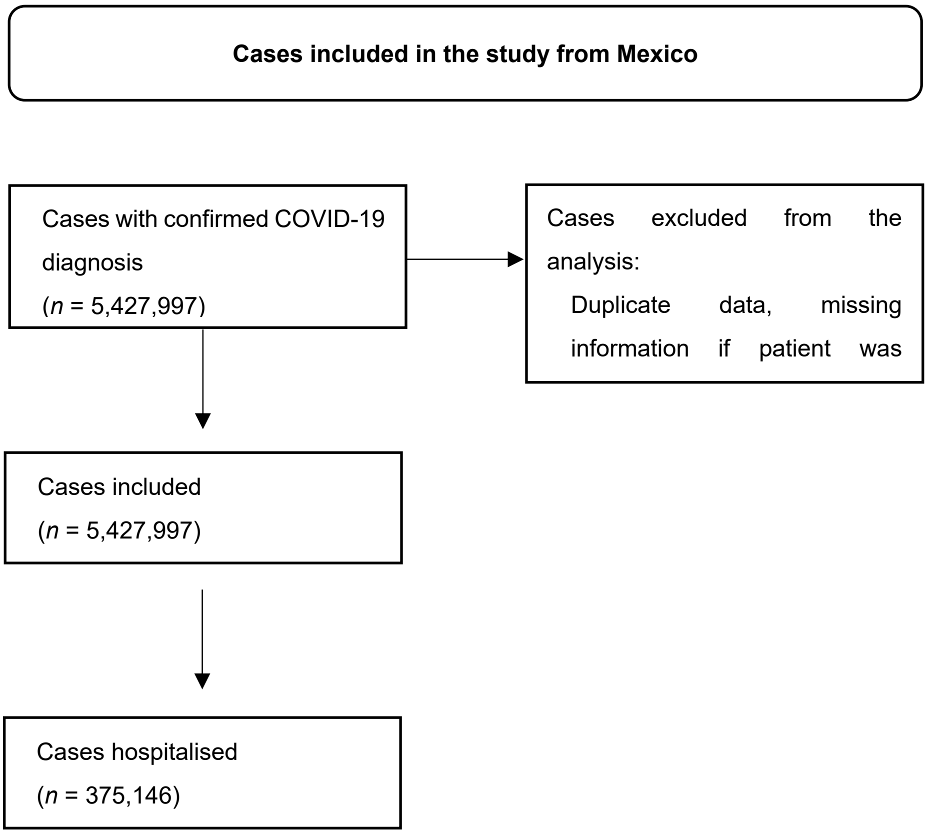

Figure S4. Patient selection in Mexico's database.

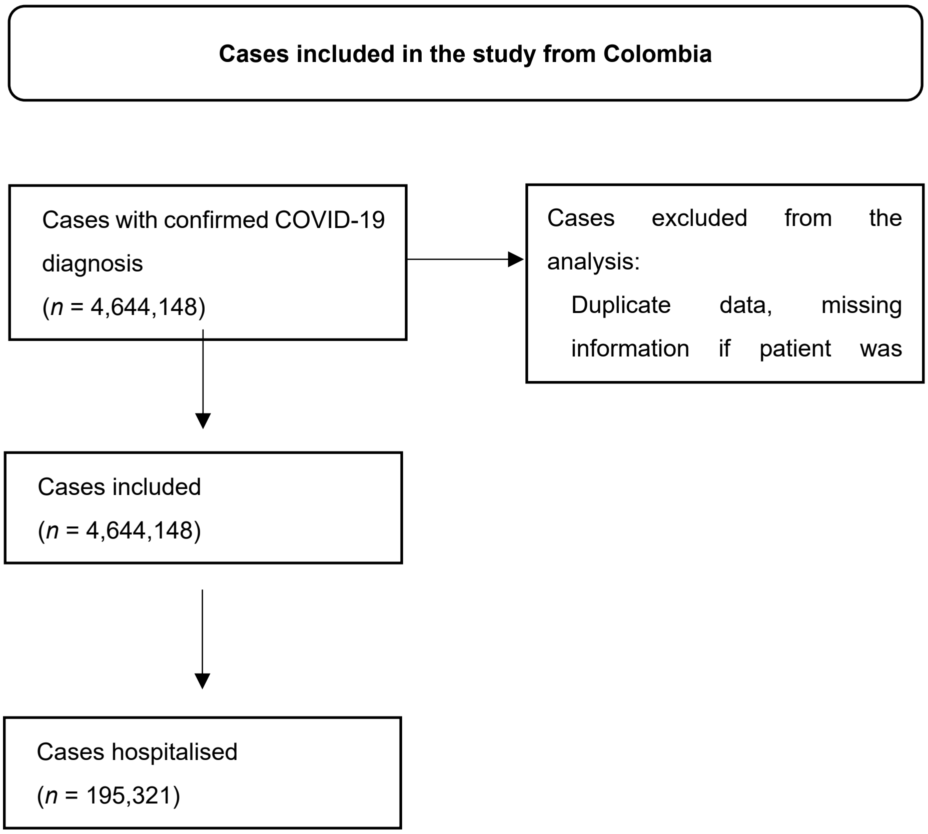

Figure S5. Patient selection in Colombia's database.

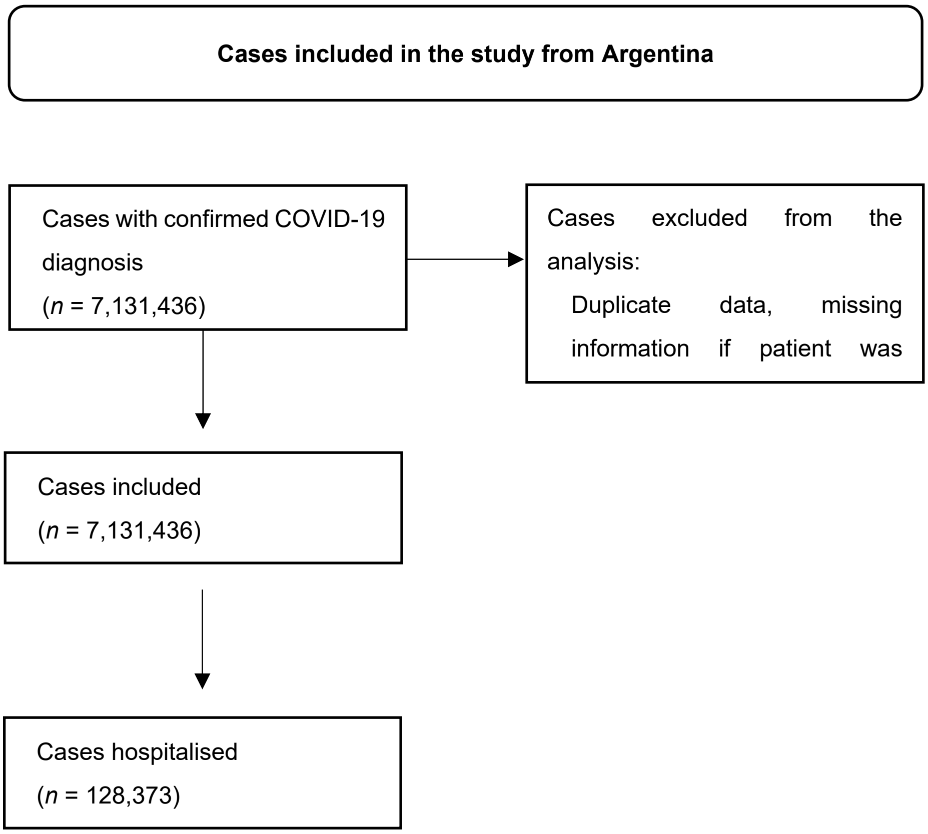

Figure S6. Patient selection in Argentina's database.

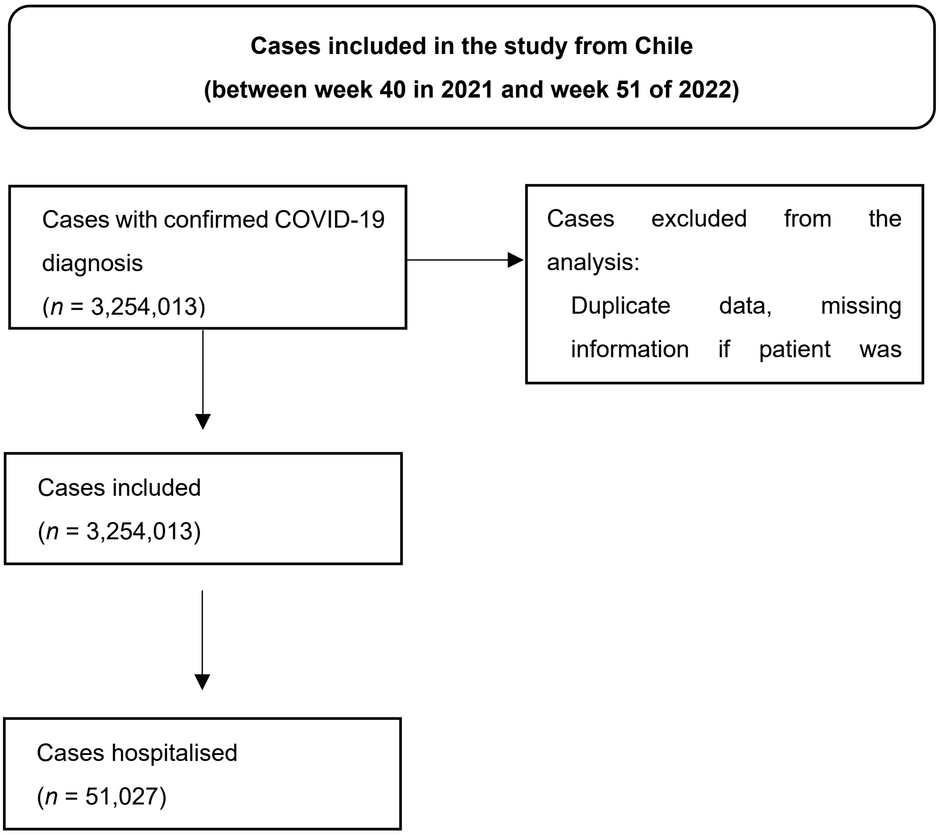

Figure S7. Patient selection in Chile's database.

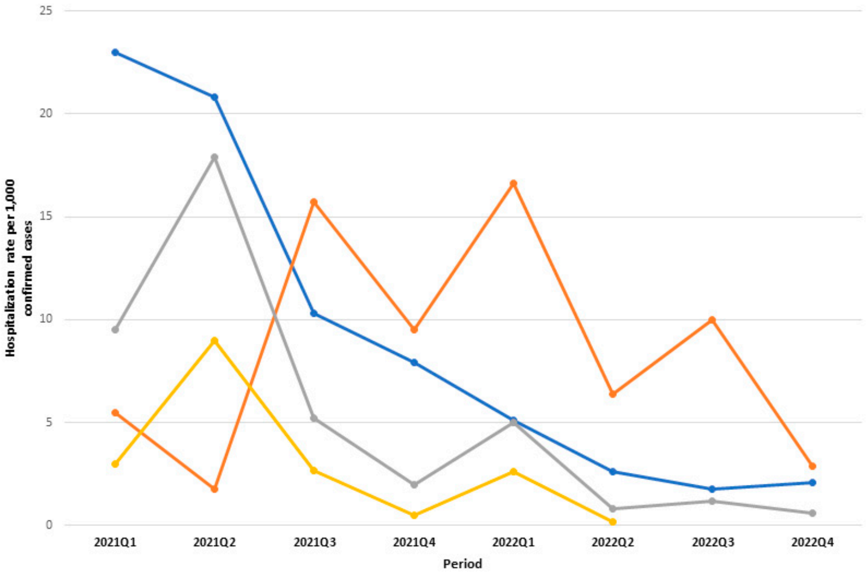

Mexico	20B/S 732 A	20B/S 732 A	20J Gamma	20J Gamma	21K Omicron	21 L Omicron	22B Omicron	22B Omicron
Brazil	20J Gamma V3	20J Gamma V3	20J Gamma V3	21J Delta	21K Omicron	21 L Omicron	22B Omicron	22B Omicron
Colombia	21H Mu	21H Mu	21H Mu	21J Delta	21K Omicron	21 L Omicron	22A Omicron	22A Omicron
Argentina	20B/S 732 A	20J Gamma V3	20J Gamma V3	21J Delta	21K Omicron	21 L Omicron	22B Omicron	22E Omicron

Figure S8. Hospitalization rate per 1000 confirmed COVID-19 cases per country, indicating the predominant SARS-CoV-2 by period.

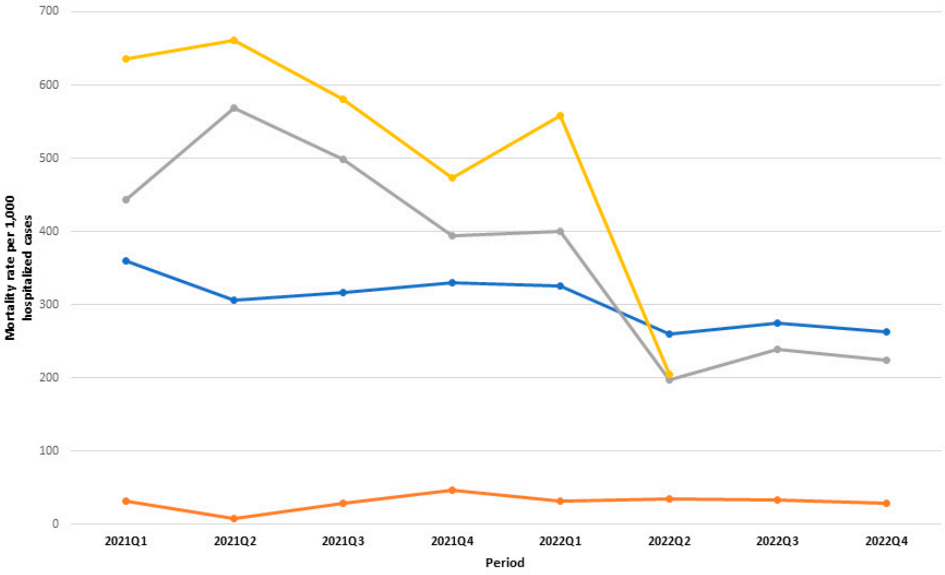

Mexico	20B/S 732 A	20B/S 732 A	20J Gamma	20J Gamma	21K Omicron	21 L Omicron	22B Omicron	22B Omicron
Brazil	20J Gamma V3	20J Gamma V3	20J Gamma V3	21J Delta	21K Omicron	21 L Omicron	22B Omicron	22B Omicron
Colombia	21H Mu	21H Mu	21H Mu	21J Delta	21K Omicron	21 L Omicron	22A Omicron	22A Omicron
Argentina	20B/S 732 A	20J Gamma V3	20J Gamma V3	21J Delta	21K Omicron	21 L Omicron	22B Omicron	22E Omicron

Figure S9. Mortality rate per 1000 hospitalized COVID-19 cases per country, indicating the predominant SARS-CoV-2 by period.
